# Optimizing Wine Production from Hybrid Cultivars: Impact of Grape Maceration Time on the Content of Bioactive Compounds

**DOI:** 10.3390/molecules31010179

**Published:** 2026-01-03

**Authors:** Anna Kostecka-Gugała, Jacek Stanula, Jerzy Żuchowski, Paweł Kaszycki

**Affiliations:** 1 Department of Plant Biology and Biotechnology, Faculty of Biotechnology and Horticulture, University of Agriculture in Krakow, al. Mickiewicza 21, 31-120 Krakow, Poland; anna.kostecka-gugala@urk.edu.pl (A.K.-G.); jacekstanula@op.pl (J.S.); 2 St. Luke’s Provincial Hospital in Tarnow, Independent Public Health Care Institution, ul. Lwowska 178a, 33-100 Tarnow, Poland; 3 Institute of Soil Science and Plant Cultivation-State Research Institute, ul. Czartoryskich 8, 24-100 Pulawy, Poland; jzuchowski@iung.pulawy.pl

**Keywords:** hybrid grapevine, antioxidant, antiradical, Johanniter, Regent, *Vitis vinifera*, polyphenol, UHPLC, ORAC, EPR

## Abstract

Wine is a rich source of biologically active compounds, particularly polyphenols, which exhibit antioxidant and antiradical properties. The objective of this study was to optimize the vinification procedures of Polish wines from the hybrid white grape cv. ‘Johanniter’ and red grape cv. ‘Regent’, grown in the temperate climate of central Europe, by applying different skin maceration times: 4, 8, 12, 16, and 20 days. The wines were compared for their basic oenological characteristics and polyphenolic (UHPLC–MS) content as well as their antioxidant (FRAP test) and antiradical (DPPH test, ORAC-fl and EPR spectroscopy) capacities. Both wines demonstrated a substantial increase in their total phenolic content and antioxidant and antiradical capacities after a 4-day maceration; further treatment did not lead to considerable enrichment in bioactive compounds. Scavenging activities against nitroxyl radicals and DPPH were divergent for the tested wines and depended on the analytical method applied, which indicated distinct molecular mechanisms. In turn, the activity of peroxyl radical scavengers, antioxidant capacity, and the total content of phenolics were higher in all the red wine samples. The antioxidant and antiradical properties of the examined wines were comparable or even exceeded those determined for most wines produced in regions with a rich winemaking tradition.

## 1. Introduction

Wine is a beverage with a profound and enduring impact on human history. In a number of regions across the globe, it fulfills a multitude of social, cultural, dietary, and religious functions. The contribution of wine to the maintenance of human health has been documented by numerous studies over the past decades. Among the bioactive wine components, polyphenolics are predominant, known to exhibit a wide range of beneficial health properties, including anticancer, antidiabetic, anti-inflammatory, cardioprotective, neuroprotective, antibacterial, antiviral, antiallergic, blood pressure-lowering, and mild anti-obesity activities as well as to have a favorable impact on the gut microbiota ([1,2,3,4] and the references cited therein). The above characteristics can be ascribed, at least in part, to antioxidant and antiradical activities, enabling the mitigation of oxidative stress. The adverse effects of this stress can be caused by many different factors, including an inadequate diet or environmental contamination. The quantity and composition of polyphenols are also pivotal determinants of wine quality, influencing its color, taste, and aroma [3].

The phenolic composition of wine and its antioxidant properties depend on several factors, including the grape cultivar, method of grape cultivation, terroir (understood as a unique combination of climatic, soil, geological, topographical conditions, sunshine, and water relations), and the vinification processes (maceration, fermentation, clarification, filtration, and aging) [5]. A substantial body of research has led to the identification of various bioactive compounds in wines, with numerous studies highlighting the beneficial impacts of phenolic compounds on human health. On the other hand, recent reports suggest that even trace amounts of ethanol can have adverse consequences, which cannot be ignored [6]. Therefore, it is difficult to predict the direction that wine research will take in the near future. Nowadays, however, some countries have gradually reduced their use of strong alcohols in favor of lighter ones, such as beer and wine. At the same time, people are becoming increasingly aware of the importance of the food they consume for their health and well-being. In this context, all efforts to optimize the production of wine that meets the requirements of a health-promoting beverage should be considered important.

Pre-fermentation maceration is a key step in the wine production process and is applied as a universal practice in the production of all wine types. It involves the incubation of crushed grapes with the pressed grape juice prior to the onset of alcoholic fermentation [7]. This process substantially influences the composition and content of bioactive compounds, which are transferred from the grape skins and seeds to the must. It causes the disruption of cell membranes, leading to an increased activity of enzymes such as pectinase and protease, and thus facilitating the extraction of polyphenols. Maceration is conducted at low temperatures (4–8 °C) to inhibit yeast growth and to prevent alcoholic fermentation, as certain phenolic compounds are effectively extracted in the absence of ethanol [8]. The use of exogenous enzymes producing pectinase, hemicellulase, and cellulase activities, in conjunction with other specialized maceration techniques, such as carbon maceration, pulsed electric field, and thermovinification, also facilitates the enhanced extraction of compounds with antioxidant properties [9].

In the present study, two hybrid grapevines commonly cultivated in Polish vineyards were selected for analysis, namely ‘Johanniter’, producing white berries, and the red-berried cultivar ‘Regent’. These hybrids are distinguished by their enhanced resistance to fungal diseases and their ability to thrive in cooler climates. In 2005, Poland was classified by the European Union Council as Wine Region A, i.e., the coldest region where vines can be grown for wine-making purposes, and which allows the sale of produced wines on the EU market (the EC Council Regulation No. 2165/2005). Despite the changeable weather conditions, exposure to frost damage, high humidity, and varied growing seasons, all posing vital challenges to the development of viticulture, some Polish wines are distinguished by their high quality, rich aroma, and elevated content of polyphenolic compounds [10]. Nevertheless, detailed research on the phenolic content and antioxidant properties of wines from the aforementioned cultivars remains limited.

The main aim of the work was to determine the effect of the maceration of hybrid grapes, that is, the white cultivar ‘Johanniter’ and the red one ‘Regent’, on the properties of the resultant wines in terms of optimizing vinification procedures. To the best of our knowledge, it is the first systematic study to reveal the influence of maceration treatment time on the composition of phenolic compounds as well as on antioxidant and antiradical capacities of the wine. The latter characteristics were assessed with several variant methods to reflect the different molecular mechanisms of the biological activity of health-beneficial compounds. Among these methods were efficient spectroscopic techniques: electron paramagnetic resonance spectroscopy (EPR) and an ORAC fluorimetric assay, which enabled the measurement of scavenging activities against different classes of radicals. In addition, the research on wine produced from the white grape cultivar ‘Johanniter’ was carried out in response to recent trends among consumers, that is, a growing interest in macerated orange wines made from white cultivars [11].

## 2. Results

### 2.1. Basic Oenological Parameters of Must and Wines

The results of the analysis of the basic parameters of musts obtained from the ‘Johanniter’ and ‘Regent’ grapes, i.e., the extract content, pH, and total acidity, *TA*_m_, are shown in Table 1.

After vinification, the following oenological parameters of wines were determined to assess the correctness of the process: pH, total acidity, alcohol (ethanol) content by volume, residual sugar content (glucose + fructose), free and total sulfur dioxide concentration, L-malic acid, and tartaric acid concentrations (Table 2).

The pH values of wines produced from the ‘Johanniter’ grapes were lower for all tested maceration times compared to those produced from the ‘Regent’ fruits. In ‘Regent’, an increase in pH values was observed after the first four days, followed by a slow, systematic decrease. In contrast, the pH of the ‘Johanniter’ wine exhibited a divergent pattern. Following an initial increase in pH values after the first four days of maceration, a subsequent, gradual increase was observed. Consequently, divergent trends in wine pH values were noticed for each cultivar between days 4 and 20 of maceration.

As regards the determination of total acidity, ‘Johanniter’ wines exhibited higher levels than ‘Regent’. This difference was observed for both non-macerated samples and wines macerated until day 16. A slight decrease in acidity along with maceration time was observed for ‘Johanniter’, whereas a drop in the ‘Regent’ acidity was pronounced only on days 4 and 8. The other oenological parameters of the wines, listed in Table 2, are addressed in Section 3. The maceration process also impacted the color, aromas, and taste of both ‘Johanniter’ and ‘Regent’ wines. Additional information regarding these characteristics has been provided in Table S1.

### 2.2. Total Phenolic Content (TPC)

**Figure 1:**
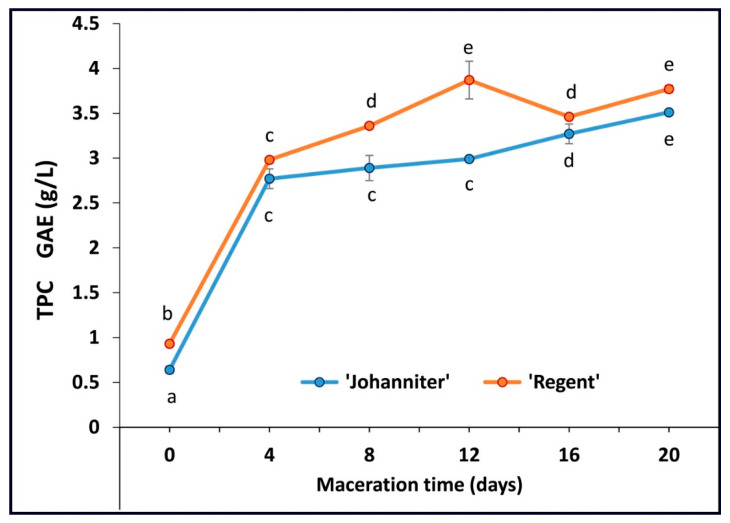
Total phenolic content, TPC, in the ‘Johanniter’ and ‘Regent’ wines, determined with the Folin assay. Different letters indicate values that differ significantly, assuming a significance threshold of *p* < 0.01.

A highly significant effect of cultivar on the TPC (*p* < 0.01) was established, with the R0 wine exhibiting a 45% higher content of phenolic compounds compared to J0 (Figure 1, Table S2). This difference was also observed for most cases of maceration. The unmacerated wines contained the lowest phenol levels, and a positive influence of maceration time on phenolic concentrations in both wines was also found to be highly significant. For both ‘Johanniter’ and ‘Regent’ wines, a dramatic increase in phenolic content was observed on day 4, i.e., a 4.33-fold increase in J4 vs. J0 and a 3.20-fold increase in R4 vs. R0, followed by a slight and still significant rise along with the subsequent maceration steps.

### 2.3. Composition of the White Wine Samples

**Figure 2:**
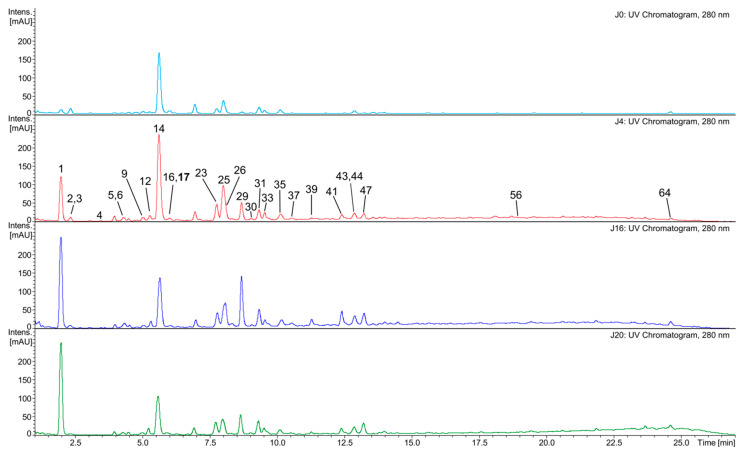
Chromatograms obtained at 280 nm for the ‘Johanniter’ wine samples: without maceration (J0, top panel, shown in light blue) and after 4-, 16-, and 20-day maceration (J4, J16, and J20; shown in red, dark blue and green on the respective panels below). The number of compounds corresponds to those listed in Table S3.

**Figure 3:**
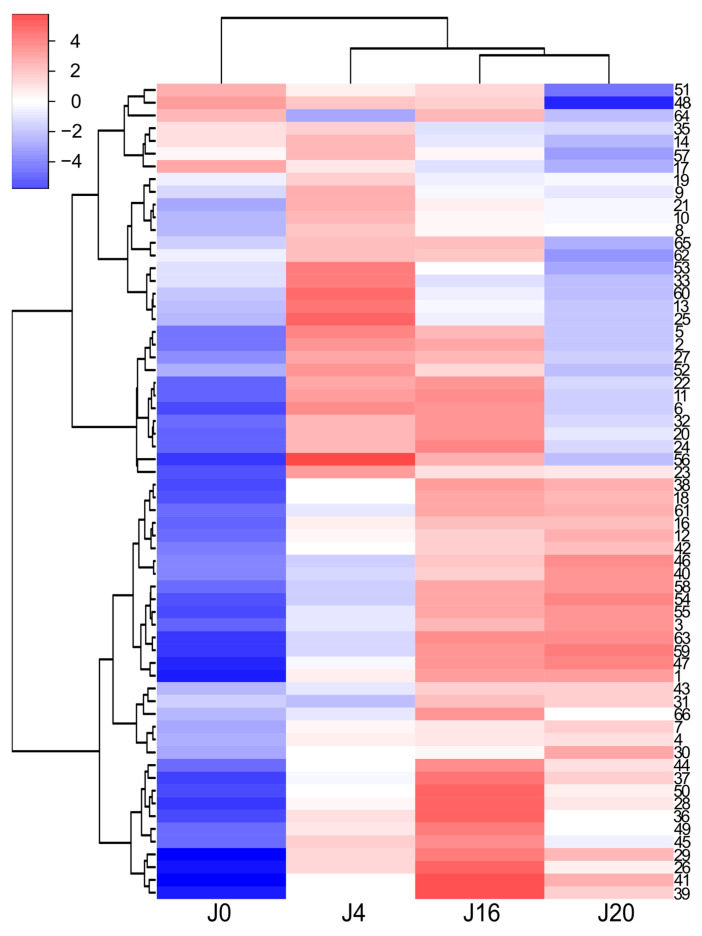
Hierarchical clustering analysis (HCA) heatmap of the relative content of 64 metabolites identified in the ‘Johanniter’ white wine upon maceration. J0, unmacerated must; J4, J16, J20, wines macerated for 4, 16, and 20 days, respectively. The number of compounds corresponds to those in Table S3.

The LC–MS analyses of the white wine samples enabled the detection of 66 phenolic and other aromatic compounds. Most of them were only tentatively identified (see Table S3 for details). The observed differences in phenolic content among J0, J4, J16, and J20 seem to be rather more quantitative than qualitative (Figure 2 and Figure 3). In general, J0 contained the lowest amounts of most phenolic compounds, compared to the macerated wine samples. Phenolic acids and their derivatives were the dominant class. Free acids comprised gallic acid, *trans*-caffeic acid, and *trans*-*p*-coumaric acid. Gallic acid was the primary phenolic constituent of J16 and J20, and the second most abundant in J4 (Figure 2), while the other two compounds were typically present in much lower amounts (except for J0). Phenolic acid derivatives comprised their tartaric acid conjugates, as well as ethyl esters and hexosides. Caftaric acid was the main phenolic compound in J0 and J4 (both samples contained similar quantities), and the second most abundant one in the remaining wine samples; in particular, coutaric acids as well as fertaric acid occurred in smaller, but still significant amounts (Figure 2). A glutathione conjugate of caftaric acid was also detected. Ethyl esters included ethyl gallate (the dominant in this group), ethyl caffeate, and ethyl coumarate. These compounds, as well as hexosides (or dihexosides) of gallic, caffeic, and coumaric acid, occurred in relatively small quantities. Flavan-3-ols, catechin, and epicatechin were other major phenolic components of J4, J20, and especially of J16. In contrast, gallocatechin and epigallocatechin were present in small amounts. Other detected flavonoids were represented by glycosides of flavonols (quercetin dihexoside, quercetin hexuronide) and dihydroflavonols (putative taxifolin deoxyhexoside), occurring in very small or trace amounts. No aglycone was detected. Proanthocyanidins were diverse, but occurred at low concentrations. This group included mainly simple B-type dimers (including procyanidin B2) as well as trimers of (epi)catechin and/or (epi)gallocatechin; a tetramer of (epi)catechin was also found. In addition, ethyl-linked dimers and trimers of (epi)catechin were also detected, as well as sulfonates of these compounds. Other phenolics or aromatic compounds, such as putative hydroxytyrosol hexoside, hydroxyphenyllactic acid, resveratrol hexoside, resveratrol, phenylalanine, putative succinyladenosine, putative tetrahydroharman-3-carboxylic acid, indolelactic acid hexoside, and indolelactic acid, occurred in small or trace amounts.

### 2.4. Composition of the Red Wine Samples

**Figure 4:**
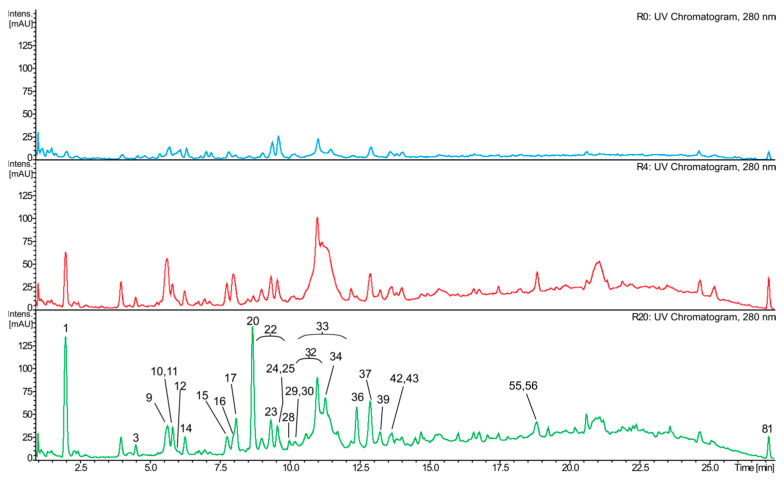
Chromatograms obtained at 280 nm for the ‘Regent’ wine samples: without maceration (R0, top panel, shown in light blue) and after 4- and 20-day maceration (R4, R20; shown in red and green on the respective panels below). The numbers of compounds correspond to those listed in Table S4.

**Figure 5:**
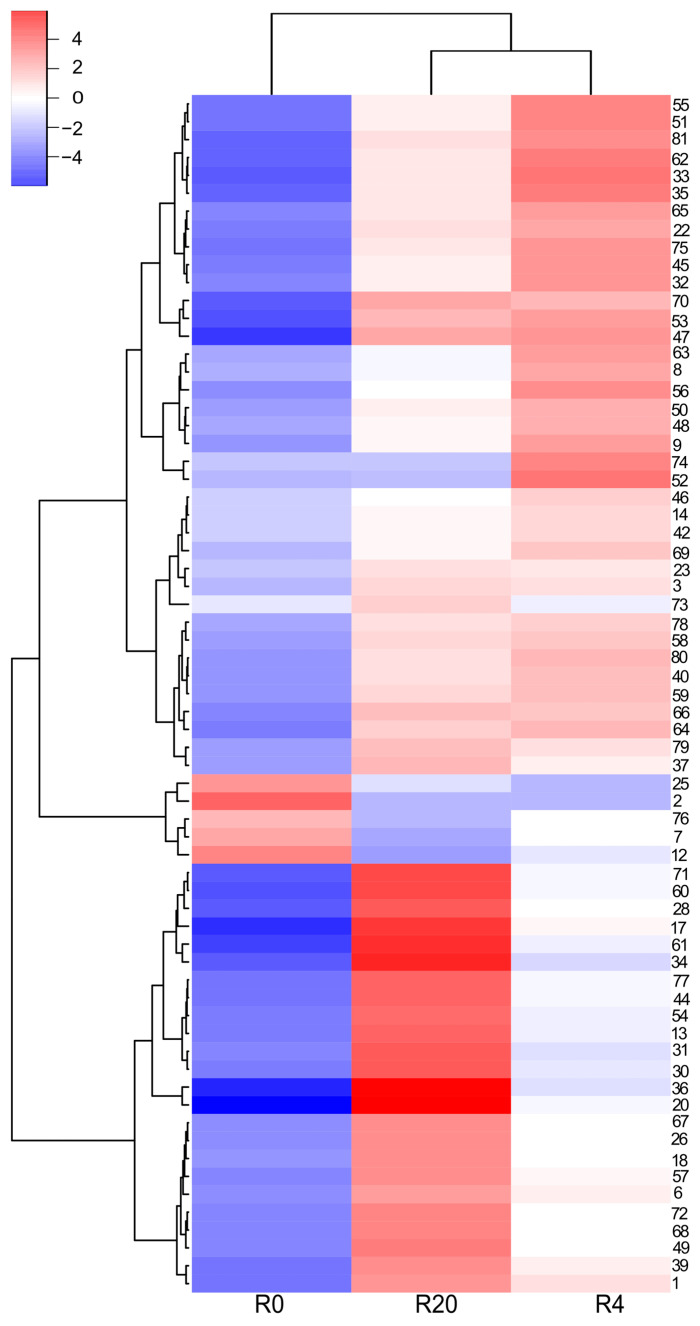
Hierarchical clustering (HCA) heatmap of the relative content of 67 metabolites identified in the ‘Regent’ red wine upon maceration. R0, unmacerated must; R20 and R4, wines macerated for 20 and 4 days, respectively. The numbers of compounds correspond to those in Table S4.

**Figure 6:**
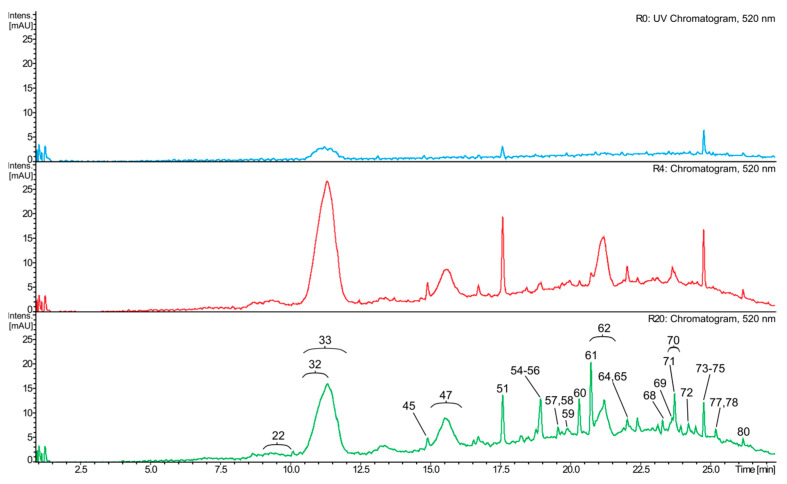
Chromatograms obtained at 520 nm for the ‘Regent’ wine samples without maceration (R0, top panel, shown in light blue) and after 4- and 20-day maceration 4- and 20-day maceration (R4, R20; shown in red and green on the respective panels below). The numbers of compounds correspond to those listed in Table S4.

Eighty-one compounds were identified in the red wine samples. Analogously to the white wine analyses, most of them were identified only tentatively (Table S4), and the observed differences in compositions of R0, R4, and R20 were essentially of a quantitative nature. Again, R0 generally had the lowest content of most phenolics (Figure 4, Figure 5 and Figure 6). The composition of the red wines was in many respects similar to that of the white wines. Gallic acid was the dominant phenolic acid in R4 and R20, while in R0, it was present only in small quantities. Other free phenolic acids comprised *trans*-caffeic acid and *trans*-*p*-coumaric acid, which were relatively highly abundant in all samples, whereas the content of a putative methylgallic acid was low. Conjugates of phenolic acids with tartaric acid were represented by caftaric, coutaric, and fertaric acids; the latter compound was present in small amounts. In addition, small quantities of glutathione adducts of caftaric acid, and a hexose conjugate of coutaric acid were also detected (Table S4). Characteristically, red wine samples contained much less *trans*-caftaric acid, as compared to the white wines (Figure 3, Figure 4, Figure 5 and Figure 6). Phenolic acids were also found as ethyl esters and hexosides. Ethyl esters comprised ethyl gallate, ethyl caffeate, and ethyl coumarate, present at relatively high amounts (Figure 4 and Figure 5); putative ethyl vanillate, ethyl syringate, and coumarate hexoside were also detected. Catechin and epicatechin occurred in all investigated samples, but only R20 contained significant amounts of these compounds, especially catechin. Only traces of epigallocatechin were detected.

As regards flavonol glycosides, minor amounts of quercetin hexuronide and syringetin hexoside were found. Dihydroflavonols were more diverse: taxifolin hexoside, taxifolin deoxyhexoside, dimethyltaxifolin dihexoside, dihydrosyringetin dihexoside, and dihydrosyringetin-coumaroyldihexoside occurred in small or trace amounts. Red wines contained significantly higher amounts of proanthocyanidins than the white ones. Diverse B-type dimers (mainly procyanidin B2), trimers, and tetramers of (epi)catechin and/or (epi)gallocatechin were also found.

The most characteristic constituents of red wines are anthocyanins and their derivatives. Among the detected anthocyanins were malvidin dihexoside (the dominant anthocyanin of all samples), malvidin hexoside, malvidin coumaroyldihexoside, malvidin caffeoyldihexoside, malvidin coumaroylhexoside, petunidin dihexoside, petunidin coumaroyldihexoside, and peonidin dihexoside. Anthocyanin derivatives were numerous (Figure 6; Table S4), and included pyranoanthocyanins (such as putative vitisin A and vitisin B), as well as adducts of anthocyanins (usually malvidin) with (epi)catechin or ethyl-(epi)catechin. Such compounds were sometimes additionally acylated with coumaric acid, and some of them were abundant, e.g., putative malvidin-hexoside-vinylphenol (most probably pigment A), malvidin hexoside pyruvic acid adduct (most probably vitisin A), or malvidin hexoside-ethyl-(epi)catechin adduct (Figure 6).

Other phenolic or aromatic compounds included putative hydroxytyrosol hexoside and hydroxyphenyllactic acid, resveratrol hexoside, resveratrol, phenylalanine, tryptophan, putative tetrahydroharman-3-carboxylic acid, putative succinyladenosine, indolelactic acid hexoside, and indolelactic acid. In addition, three sulfonated compounds were detected, probably malvidin and its glycosides. All these substances occurred in small or trace amounts.

### 2.5. Antioxidant Capacity (FRAP)

**Figure 7:**
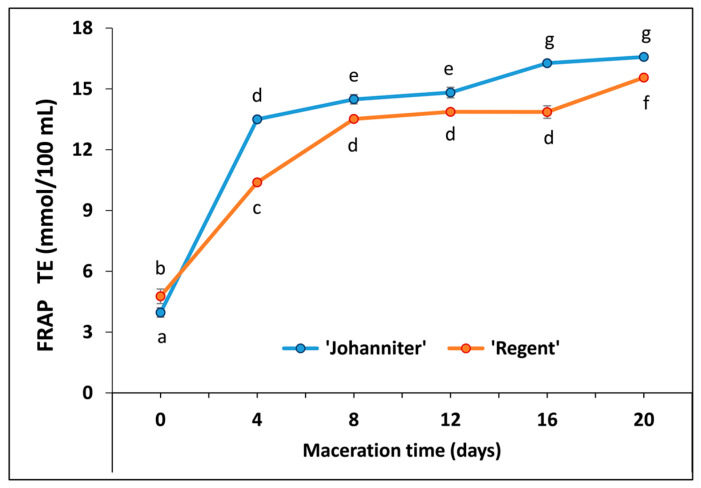
Antioxidant capacity of the ‘Johanniter’ and ‘Regent’ wines determined by the FRAP assay. Different letters indicate values that differ significantly, assuming a significance threshold of *p* < 0.01.

Antioxidant capacities determined with the FRAP assay were different for the tested grape cultivars with high statistical significance (*p* < 0.01) (Figure 7, Table S5). Maceration proved crucial for enhancing the antioxidant capacities of both wines. The effect was the most pronounced on the fourth day, when a 3.40-fold increase was recorded for J4 and a 2.18-fold increase for R4. A steady upward trend was observed in the following days. The macerated white wine produced from ‘Johanniter’ exhibited higher antioxidant capacities compared to the macerated ‘Regent’ wine, and this discrepancy was found to be significant at each of the analyzed maceration times.

### 2.6. Antiradical Capacity (DPPH, Colorimetric Test)

**Figure 8:**
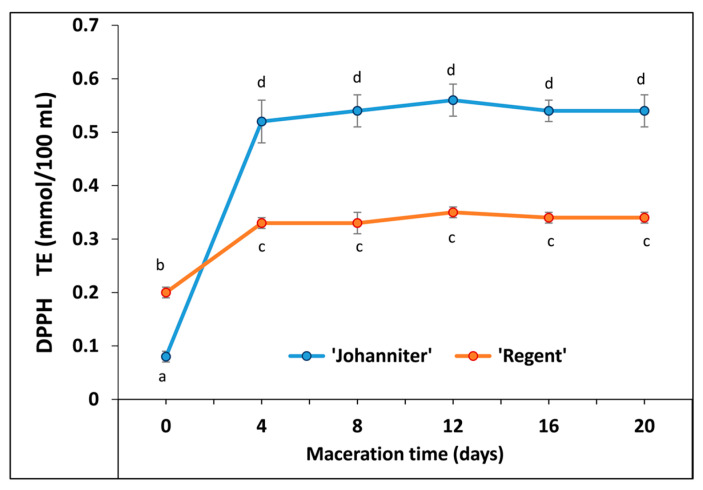
Antiradical capacity of the ‘Johanniter’ and ‘Regent’ wines determined by the DPPH colorimetric assay. Different letters indicate values that differ significantly, assuming a significance threshold of *p* < 0.01.

Highly significant (*p* < 0.01) differences between cultivars were noted for antiradical capacity measured with the DPPH colorimetric assay (Figure 8, Table S6). Unmacerated wines revealed the lowest antiradical capacities; however, the value determined for R0 was 2.5-fold higher than for J0. On the fourth day of maceration, the antiradical capacity recorded for ‘Johanniter’ increased dramatically by 6.5-fold, markedly exceeding the values observed for the red wine produced from ‘Regent’. Subsequent processing steps did not influence the measured parameters of either wine cultivar, while the observed differences between wines persisted along with the prolonged maceration.

### 2.7. Oxygen Radical Absorbance Capacity (ORAC-Fl)

**Figure 9:**
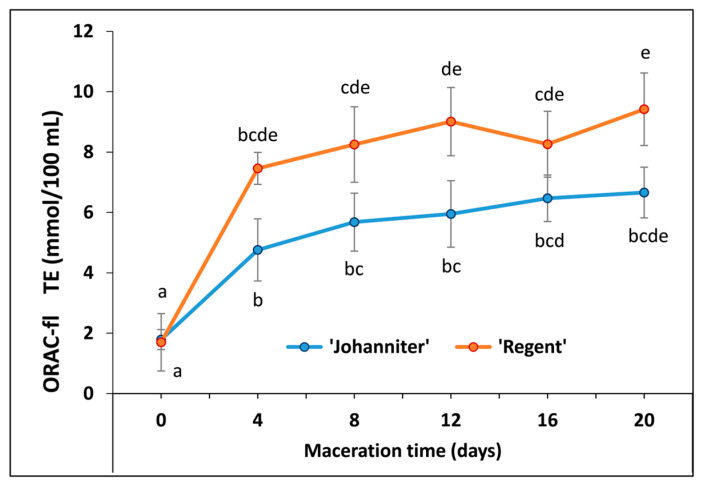
Antiradical capacity of the ‘Johanniter’ and ‘Regent’ wines determined by the ORAC-fl assay. Different letters indicate values that differ significantly, assuming a significance threshold of *p* < 0.01.

The peroxyl radical scavenging potential, determined with ORAC-fl showed similar antiradical capacities for both unmacerated wines. The maceration process led to a considerable increase in antiradical capacity in all samples, and the most dramatic effect was visible on day 4 of the must processing. In general, ‘Regent’ exhibited higher values compared to ‘Johanniter’; however, detailed statistical analysis showed that when each maceration time was considered separately, highly significant (*p* < 0.01) differences between wines of the two cultivars occurred only on day 12 (Figure 9, Table S7).

### 2.8. Antiradical Capacity Measured with EPR

**Figure 10:**
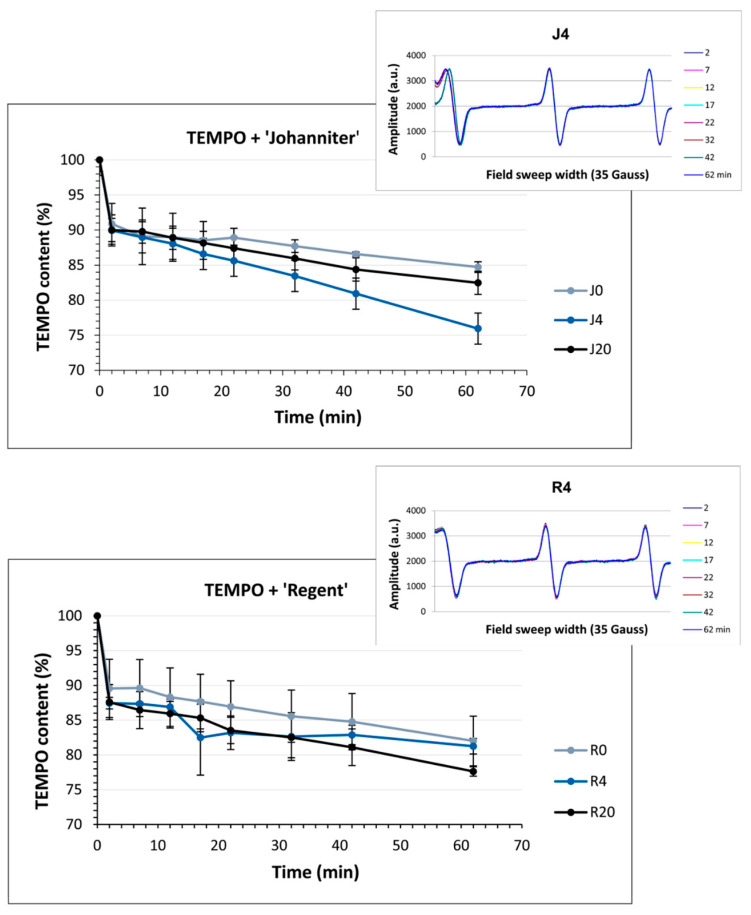
Kinetic curves of TEMPO radical quenching by antiradical compounds present in the ‘Johanniter’ (**top**) and ‘Regent’ (**bottom**) wines as measured with EPR spectroscopy. Inserts show exemplary raw EPR signals of TEMPO obtained for the samples J4 (**top**) and R4 (**bottom**), recorded after 2, 7, 12, 17, 22, 32, 42, and 62 min of reaction with the radical.

TEMPO is a water-soluble nitroxide that acts as a stable spin label. Its incubation with the antiradical compounds occurring in wines led to a reduction in TEMPO, especially in the initial minutes of the reaction. This was observed as an amplitude decrease in the EPR signal (Figure 10). Approximately 20% of the radical was reduced within 62 min of the experiment. It is noteworthy that the strongest antiradical potential was documented after incubation of TEMPO with the sample J4.

**Figure 11:**
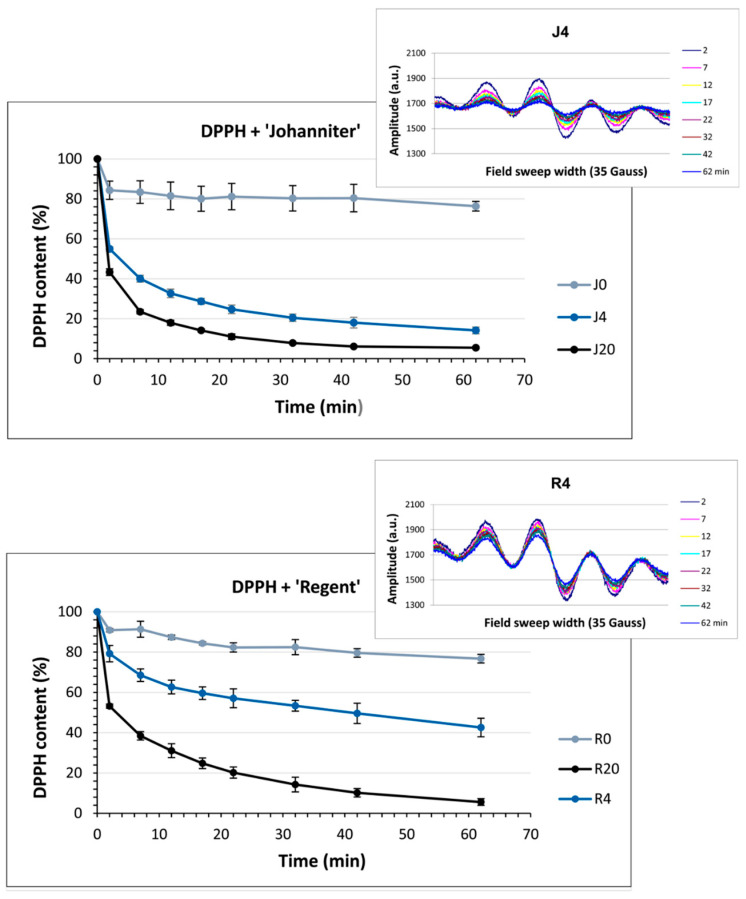
Kinetic curves of DPPH radical quenching by antiradical compounds present in ‘Johanniter’ (**top**) and ‘Regent’ (**bottom**) wines as measured with EPR spectroscopy. Inserts show exemplary raw DPPH signals obtained for the samples J4 (**top**) and R4 (**bottom**), recorded after 2, 7, 12, 17, 22, 32, 42, and 62 min of reaction with the radical.

The use of an alcohol-soluble DPPH spin label revealed different radical reduction kinetics when compared to TEMPO (Figure 11). Antiradical compounds present in unmacerated wines J0 and R0 reduced DPPH most efficiently during the initial stages (2 min) of incubation. Then, a gradual, slower decrease in the EPR amplitude was observed for both wines, which reached about a 20% reduction rate after 62 min. In turn, macerated wines caused a much more pronounced decrease in the DPPH radical signal. Moreover, prolonged maceration resulted in an elevated amount of antiradical compounds capable of DPPH reduction. In consequence, less than 10% of the DPPH radical remained in the samples of both the white and red wines macerated for 20 days. Interestingly, J4 revealed a considerably higher DPPH-antiradical activity compared to R4.

## 3. Discussion

In this study, six wines were produced from the hybrid grape cultivar ‘Johanniter’ and another six from ‘Regent’, applying different must maceration times, i.e., 0 (no treatment), 4, 8, 12, 16, and 20 days. Prior to maceration, the ‘Johanniter’ fruit produced a white wine with pH of 3.23, while the ‘Regent’ berries yielded a red wine exhibiting pH of 3.75. These values are in accordance with the typical ranges reported for such types of wines produced in central Europe [12]. In general, white wines have higher concentrations of organic acids, which is reflected by their lower pH values. In addition, wines originating from grapevines cultivated in cool climates exhibit higher acidity levels compared to those from warmer wine regions [13].

For acidity evaluation, the *TA*_w_ values obtained for ‘Johanniter’ tended to decrease along with maceration time, while the pH value increased. This relationship was confirmed in the literature [14]. A decline in *TA*_w_ is typically observed during the fermentation process and is attributed to the precipitation of potassium hydrogen tartrate (KHT), which is less soluble in ethanol solutions. However, for wines with a pH below 3.65, KHT precipitation is minimal because it remains in solution, either as a dissolved salt or free tartaric acid [15]. Therefore, it can be assumed that the *TA*_w_ decrease observed for ‘Johanniter’ resulted from malolactic fermentation (MLF) or, less likely, from a high content of potassium ions in the solution [13]. Conversely, in wines with a pH above 3.65, KHT precipitation leads to an increase in pH, which is accompanied by a simultaneous decrease in total acidity, as the bitartrate anion (HC_4_H_4_O_6_^–^) is removed from the solution. This causes a shift in the equilibrium of tartaric acid, which reacts with protons, thereby reducing their concentration [16]. Accordingly, for the ‘Regent’ wine, a significant drop in *TA*_w_ was noted at the initial stage of maceration (*TA*_w_ of 8.19 for R0 and 7.24 for R4), although further processing yielded no significant changes.

It is widely accepted that the presence of organic acids in wine provides a refreshing sensation, helping to balance fruity and sweet flavors. In wines with low acidity, these flavors may be perceived as less intense [17]. In addition, wine acidity can modify the perception of alcohol, making the taste of the beverage sweet rather than burning [18]. Moreover, ensuring the right level of acidity is considered essential for maintaining wine’s microbiological stability and increasing the effectiveness of SO_2_ use. It is also crucial to obtain the desired sensory properties during the aging process [19]. The analysis of L-malic and tartaric acid concentrations showed that the red wine ‘Regent’ subjected to at least four days of maceration underwent spontaneous malolactic fermentation (MLF) simultaneously with alcoholic fermentation. This is also evidenced by an accompanying increase in pH and a decrease in total acidity. Interestingly, MLF did not occur in white wine ‘Johanniter’, despite identical vinification conditions. These differences might be explained by the lower pH level in the white must relative to the red one (3.26 and 3.62, respectively), which could have inhibited the activity of lactic acid bacteria and thus prevented MLF from being initiated. An inhibitory action of free sulfuric acid on MLF, as described by Ribéreau-Gayon et al. [13], can be neglected since the concentration of free and total SO_2_ in all wine samples remained relatively low (Table 2).

MLF was shown to substantially contribute to the antioxidant activity. This fermentation process is attributed to the enzymatic activity of bacteria capable of hydrolyzing phenolic acid esters and glycosides, thereby releasing their acid or aglycone forms [20]. It is thus reasonable to assume that, besides progressive enrichment in other antioxidants along with maceration (especially anthocyanins, see below), efficient MLF observed for the ‘Regent’ samples resulted in the considerably higher final antioxidant capacity values of these wines. Total phenolic content (TPC), determined by maceration time and expressed as GAE, for the case of ‘Regent’ wines ranged from 0.93 g/L (R0) to the maximum value of 3.87 g/L achieved after 12 days of maceration. For ‘Johanniter’, the initial TPC values were lower (0.64 g/L, J0), and then tended to increase up to 3.51 g/L after 20 days of maceration. It is worth emphasizing here that the initial maceration step (4 days) resulted in the most dramatic increase in TPC for both wines tested. Furthermore, maceration resulted in considerably higher TPC values compared to the results of the few available studies on the same wine types produced in Poland. Kapusta et al. [21] and Wojton [22] analyzed TPC in both ‘Johanniter’ and ‘Regent’. In the first study, the authors reported respective values of 0.098 and 1.86 g/L [as GAE units], whereas the second work established the respective TPC levels at 0.149 and 1.07 g/L. Other papers examined the ‘Regent’ wine only and revealed the following TPC values: 1.7–3.0 g/L [23], 0.99 g/L [24], and 1.16–3.17 g/L [25]. The TPC levels obtained in our study after wine maceration should also be considered high compared to data reported for other wine types. For example, the analyses of wines produced from the most famous grape cultivars grown in regions of warmer climate (Bordeaux, France) showed TPC values ranging from 1.57 to 3.1 g/L for 24 different samples of ‘Cabernet Sauvignon’, and from 1.24 to 2.54 g/L for seven ‘Merlot’ wines [26].

It is worth noting that maceration of white wines from the ‘Johanniter’ grapes resulted in particularly high TPCs, making them similar to the values obtained for the red ‘Regent’ macerated wine (cf. the lack of statistical differences between sample pairs: J4, R4, and then J16, R16; see Table S2). According to data from Stratil et al. [27], the TPC of typical white wines usually ranges from 0.90 to 0.166 g/L [GAE], while red wines contain significantly higher amounts of phenols, ranging from 0.874 to 1.973 g/L. The aforementioned difference in phenolic content between white and red wines is primarily attributable to the distinct winemaking processes employed in their production. Red wine requires longer maceration, which allows phenolic compounds to be extracted from the skins, seeds, and stems of grapes. White wines, in turn, are most often produced by fermenting the juice itself immediately after pressing, or after a short maceration at low temperature. The main purpose of the latter process is to increase aromatic complexity rather than to extract phenols. During maceration, phenolic compounds, in particular hydroxycinnamic esters and flavanols (e.g., catechin, epicatechin), are converted into reactive orthoquinones. These compounds contribute to the oxidative browning of wine, which is generally undesirable in the production of wines from white grape cultivars and therefore prolonged maceration is relatively rare [28].

As regards wine beneficiation with maceration, Gómez-Miguez et al. [14] demonstrated that anthocyanins, which are glycosides that are stable at lower temperatures, were extracted more efficiently than other phenolics. For the case of hydroxycinnamic acids and flavonols, the authors found the levels of these compounds to be comparable to those of anthocyanins; however, the extraction rate was lower, which was attributed to poorer water solubility. An increase in the maceration time resulted in a notable elevation in the concentration of catechins, epicatechin, and tannins, although the extraction rate tended to decline, indicating the possibility of saturation of the aqueous environment [29]. The processes of maceration that transpire during fermentation and then follow this stage are of paramount significance in the vinification of red wines. Maceration duration can vary widely, ranging from a few days to several weeks. For white and rosé red wines, it is notably brief and typically does not exceed 24 h.

However, for the case of Georgian Qvevri white wines, long maceration of the skins often results in phenolic levels similar to those found in red wines. These wines (both white and red) are produced by macerating uncrushed grapes in qvevris, large clay vessels buried in the ground. Wine production requires prolonged contact between the extract and the skins, both during and after fermentation. In addition, micro-oxygenation, facilitated by the porous walls of the qvevri, plays a key role in fermentation, influencing the phenolic profile and stability of the wine. After fermentation, the wine remains in contact with the skins at 12–14 °C. After one or two months, the solid parts of the grapes are pressed, and the wine is returned to the qvevri, where it continues to mature [30]. In the case of orange wines produced from white grapes, the colors of which are similar to our macerated ‘Johanniter’ wines, the main purpose of maceration is to intensify the extraction of compounds that are less soluble in water. According to the research conducted by Bautista-Ortín et al. [31], when maceration occured before fermentation, the extraction of water-soluble compounds increased. These compounds include anthocyanins (present in the skins) and low molecular weight flavonoids (found mainly in the seeds). In contrast, higher molecular weight flavanols and flavonols from grape skins, as well as phenolic acids, are more soluble in alcohol than in water. However, it was shown that some simple phenolic compounds, such as gallic acid and protocatechuic acid, also required longer maceration to increase their concentration in wine [32].

Bene and Kállay [33] examined the total polyphenol content in white wine produced from ‘Zéta’ grapes harvested from a single vineyard. The wines were compared in terms of production methods—both traditional and qvevri. It was found that TPC in macerated wines was 1.63 g GAE/L, while in traditional wines it was about five times lower, that is 0.36 g/L. The combination of maceration with grape drying was shown to be effective in terms of increasing the concentration of bioactive compounds in wine. A study conducted by Kowalczyk et al. [34] analyzed the TPC and antioxidant potential of three wines produced using different technologies: grape drying, grape drying together with seven-day maceration, and wine refermentation using dehydrated grapes. The wine obtained by drying the fruit and seven-day maceration showed the highest level of polyphenols and antioxidant activity. As other researchers also documented [14], maceration considerably influenced the extraction of phenolic compounds into wine. The authors analyzed nine different white wines, each of which was subjected to a different contact time between the skins and the extractant (2, 4, 6, 8, 12, 18, and 24 h). The results showed that prolonged maceration time, defined as exceeding 12 h, led to a significant increase in the concentration of phenolic compounds in the finished wine compared to unmacerated ones. In another study, Alencar et al. [35] analyzed the effect of maceration on the polyphenol content and antioxidant activity of wines obtained from the ‘Syrah’ cultivar grown in a warm climate. Their findings proved that maceration promoted an increase in phenolic compounds until day 15, while the main extraction of anthocyanins took place until day 20. The total concentration of phenolic compounds stabilized on the 20th day and then remained constant until the end of treatment. Ružić et al. [36] examined the phenolic composition and antioxidant potential of white wines from the ‘Malvazija Istarska’ cultivar before and after prolonged maceration (14–21 days). The produced wines were characterized by a considerably higher total phenol content and almost six-times-higher average antioxidant capacity, which was a result comparable to that of some red wines. On the contrary, Gómez-Míguez et al. [14] observed a decrease in the content of polyphenols, in particular flavonoids, in white wines subjected to prolonged maceration, with a simultaneous increase in the content of non-phenolic compounds.

Casassa et al. [37] examined the effects of prolonged one- or six-month maceration on wines produced from ‘Pinot Noir’ and ‘Zinfandel’ grapes. Six-month maceration led to a significant reduction in the content of anthocyanins and their derivatives—by 53% in ‘Pinot Noir’ wines and 63% in ‘Zinfandel’ wines, respectively—compared to the control, unmacerated samples. At the same time, tannin content increased significantly: 13-fold in ‘Pinot Noir’ and 1.6-fold in ‘Zinfandel’. These results indicate that beyond 30 days of maceration, further extraction of phenolic compounds became ineffective. The authors concluded that prolonged maceration leads to a reduction in the content of valuable polyphenolic compounds, while simultaneously increasing tannin concentration, which may ultimately compromise the sensory quality of the wine. Francesca et al. [38], while examining the effect of extended maceration on the polyphenolic composition and antioxidant activity of the red wine from ‘Aglianico di Taurasi’ grapes, demonstrated that wines macerated for 40–50 days had the highest content of phenolic compounds and maximum antioxidant capacity. The dominant phenolic compounds were benzoic and cinnamic acids, as well as catechins. Considering white wines subjected to extended maceration, it was found that three main groups of phenols dominated, i.e., flavonoids, as well as hydroxycinnamic and benzoic acid derivatives [3]. The total flavonoid content in macerated wines was seven times higher than in the other samples.

A more thorough analysis of phenolic compounds present in the tested wines was carried out with the LC–MS technique (Tables S3 and S4). Phenolic acids and their derivatives were predominant in the ‘Johanniter’ white wine samples. Among the identified compounds were gallic acid, caffeic acid, *p*-coumaric acid, and their ethyl esters, as well as caftaric acid, coutaric acid, fertaric acid, and their *cis* isomers, as well as a glutathione conjugate of caftaric acid and a coutaric acid conjugate with hexose. The presence of these phenolics in white wines is well documented in the literature [39,40,41,42]. Among the white wines, J4 had the highest content of i.a. caftaric acid, coutaric acids, piceid, as well as flavonol and dihydroflavonol glycosides. The longer maceration contributed to the further increase in the content of most of the remaining phenolics, which is visible especially in the case of the wine J16. Here, it should be noted that the 20-day maceration of ‘Johanniter’ grapes, in combination with a low pH level, resulted in instability and cloudiness during wine storage. This fact made us examine an additional clear sample, J16, employing LC–MS analysis. A vast body of literature suggests that sodium potassium tartrate frequently precipitates from young wine, while phenol precipitation is less probable [43]. However, our analysis revealed a reduction in the pool of phenolics, and further research is necessary to ascertain the cause of this phenomenon. It can be posited that wine that has undergone extended maceration may require additional stabilization. This emphasizes the advantages of limiting this process to a specific time frame. J16 had the highest levels of catechin, epicatechin, and proanthocyanidins (Figure 2 and Figure 3). Quite similar results were obtained for wines with longer maceration times (from 30 to 120 days), made from ‘Minutolo’ and ‘Verdeca’ [44], or ‘Graševina’ grapes [45], which had much higher levels of flavonoids (flavanols) and proanthocyanidins, as compared to control wines. The macerated ‘Graševina’ wine was also shown to have a significantly higher content of gallic and caftaric acid. The detected glutathione conjugate is most probably 2-S-glutathionyl caftaric acid, known as the Grape Reaction Product [46]. Catechin and epicatechin were the main flavan-3-ols of the white wine samples. Proanthocyanidins, present in small amounts, were represented mainly by B-type dimers and trimers of (epi)catechin and/or (epi)gallocatechin, which is quite consistent with the available literature data on flavan-3-ols and proanthocyanidins of white wines [3,40,47]. Ethyl-linked dimers and trimers of (epi)catechin were also detected. Such compounds were previously found in red wines, red grape seeds, or were synthesized in environments that mimicked the chemical conditions found in wine [46,48,49,50]. The white wine flavonoids were represented by glycosides of quercetin, and putative taxifolin occurred in very small quantities; all of these compounds were earlier reported in publications on white wines [3,40,47,51].

In the red wine samples, apart from the phenolic compounds identified in ‘Johanniter’, which are in line with the literature data [39,42,52], additionally, glycosides of putative syringetin, dihydrosyringetin, and dimethyltaxifolin were detected. Similar glycosides of quercetin, syringetin, taxifolin, and dihydrosyringetin were earlier detected in grape wines and/or grapes [52,53,54]. Flavan-3-ols and proanthocyanidins from the red wines were quite similar to those described in the literature [50,54,55]. It is well known that a specific feature of red wines is the presence of anthocyanins and their derivatives. The tested ‘Regent’ samples contained several anthocyanins, mainly malvidin glycosides, as well as small amounts of petunidin and peonidin glycosides. Similar compounds were often reported by other authors, namely non-acylated and acylated glucosides or diglucosides of malvidin, as well as peonidin, petunidin, delphinidin, and cyanidin [55,56,57,58]. The dominant anthocyanin identified in this study was malvidin dihexoside (most probably malvin, malvidin 3,5-*O*-diglucoside). Cultivars of *V. vinifera* tend to contain only small or trace amounts of diglycosidic anthocyanins [55,56,57,58] while the presence of such compounds seems to be a characteristic feature of some hybrid red grape cultivars, where malvin is a major anthocyanin [21,56,58,59]. The ‘Regent’ wines also contained diverse pyranoanthocyanins, and adducts of anthocyanins with (epi)catechin or ethyl-(epi)catechin, similar to those reported by others [60,61].

It was observed that the highest level of most anthocyanins and pyranoanthocyanins was achieved in R4. The longer maceration usually decreased the content of these compounds. Similar results were achieved by Ferrero et al. [62], who detected the highest level of anthocyanins in ‘Nebbiolo’ wines on the 4th day of maceration at 24 °C, and on the 2nd day of maceration at 29 °C. The longer maceration time (up to 12 days) led to a gradual decrease in the level of these compounds. Another research group obtained similar results for wines made from ‘Karaoglan’ grapes [63]. In the case of gallic acid, catechin, epicatechin, proanthocyanidins, and adducts of anthocyanins with (epi)catechin (including the ethyl-bridged ones), the highest concentration of these compounds was detected in R20 (Figure 5 and Figure 6). A gradual increase in the content of catechin, epicatechin, as well as gallic acid was also observed in ‘Cabernet Sauvignon’ wines, during the 21-day maceration experiment [64]. The results of some other experiments are also in line with our data [62,63].

Several sulfonated compounds were also detected; sulfonated derivatives of ethyl-bridged dimers of (epi)catechin were found in the white ‘Johanniter’ wines. Sulfonated catechin and dimeric proanthocyanidins were previously reported for red wines [65,66]. They were not detected in the analyzed ‘Regent’ samples, which instead contained putative sulfonate derivatives of malvidin hexoside and malvidin. According to the literature data [67], the product of bisulfite addition to malvidin 3-*O*-glucoside, supposed to occur in some SO_2_-conserved food products and wine, had a different formula (one oxygen atom less) than the compounds detected in this study. However, this compound was not isolated from wine, but was synthesized from malvidin 3-*O*-glucoside and NaHSO_3_, and it is still possible that wines may contain alternative forms of sulfonate derivatives of malvidin hexosides and malvidin as well. As expected, the analyzed wines also contained small amounts of resveratrol, piceid, and their isomers. In addition, small or trace quantities of phenylalanine, tryptophan, putative hydroxytyrosol hexoside and hydroxyphenyllactic acid, phenylalanine, indolelactic acid hexoside and indolelactic acid, putative tetrahydroharman-3-carboxylic acid, and succinyladenosine were found. Most of these compounds (except for tetrahydroharman-3-carboxylic acid, which was tentatively identified on the basis of its determined formula as well as fragmentation spectra) were previously reported to occur in wines or grapes [66,67,68,69,70]. The detected putative succinyladenosine was probably produced by yeasts [71].

Two spectrophotometric assays (FRAP and DPPH) and the hydrophilic version of the ORAC-fl fluorimetric test were used to establish the antioxidant and antiradical properties of the wines (see Section 4.5.4, Section 4.5.5 and Section 4.5.6 for detailed descriptions). The results of these analyses were described as antioxidant/antiradical capacity values that reflect the effect of various antioxidants on the course of a specific reaction. It should be noted, however, that each test actually detects a reaction product formed by a different mechanism. Therefore, even though the results of all three applied methods were expressed using equivalents of Trolox, a highly stable model antioxidant in both aqueous and alcoholic solutions, they cannot be compared directly and should rather be considered as complementary approaches.

The FRAP assay is based on the measurement of the reducing properties of antioxidants, i.e., their electron-donating capacity. The Fe^3+^ reduction reaction is carried out by single-electron transfer (SET). The DPPH antiradical capacity test, in turn, uses a stable organic radical that, in theory, can interact with antioxidants via the following mechanisms: SET, ET-PT (electron transfer followed by proton transfer), HAT (proton transfer), or SPLET (sequential proton loss electron transfer). The reaction mechanism depends on the pH and polarity of the environment, as well as the structure and concentration of the antioxidant [72]. In our case, calculations performed for both wines showed that mixing diluted ‘Johanniter’ and ‘Regent’ wines in the appropriate proportions with an ethanolic DPPH solution (see Section 4.5.5) yielded solutions with an acidic pH of approximately 5. At this pH, phenols mainly occur in their neutral form (PhOH) because the pKa constant of most phenols is between 9 and 10. Consequently, under these conditions, in a protic (ethanol:water) environment, reactions involving the HAT mechanism are more probable. However, it is important to note that the literature on this subject is inconsistent and sometimes unclear regarding the mechanism of this reaction [72]. Phenol molecules have certain features that may facilitate HAT. The low bond dissociation energy (BDE) of the O–H bond and the presence of electron-donating groups (–OH and –OCH_3_) in the aromatic ring are key factors in the HAT process. These groups stabilize the phenoxyl radical through a resonance mechanism and facilitate hydrogen atom transfer under acidic conditions. High number of hydroxyl groups located in optimal positions, as well as the coupling of aromatic rings (as seen in flavonoids), are also favorable features [73].

ORAC-fl assesses the ability of antiradical compounds such as phenols to scavenge peroxyl radicals (ROO•) while the pH of the reaction environment is raised to 7.4. These radicals are formed in situ as a result of the thermal decomposition of AAPH in an aqueous solution [74]. They oxidize fluorescein, causing a decrease in fluorescence signal intensity, while the activity of antiradical substances has a fluorescein-protective effect. Unlike in the DPPH reaction, phenols act primarily due to the nature of the radical and the environment. Unlike in the DPPH reaction, phenols act primarily due to the nature of the radical and the environment. Peroxyl radicals are smaller and more reactive than the stable DPPH model radicals [75], and therefore, they are more accessible to the phenol –OH groups. In addition, in an aqueous environment with a pH between 7 and 7.4, nearly all (99%) phenols remain undissociated, which suggests that the SET mechanism is essentially non-existent. The remaining dissociated phenols, i.e., phenolates (PhO^–^), constitute less than 1% of the total pool and are shown to affect the rate of the HAT reaction, commonly considered to be dominant [74,76]. The contribution of phenolates can be attributed to their considerably lower BDE O–H value compared to PhOH when a proton is added to an anion. Consequently, phenolates exhibit increased reactivity with peroxyl radicals. It is noteworthy that while most of the references cited above indicate that phenols react with ROO• via the HAT mechanism, some studies, including that of Zhang et al. [77], suggest that accurate calculations of BDE, ionization potential, electron transfer enthalpy, and proton affinity imply that SPLET is the principal mechanism for reactions with flavonoids. In the context of ORAC-fl measurements of wine samples, polyphenols with multiple –OH groups in an ortho/para arrangement exhibit the highest reactivity. These compounds are characterized by their low BDE–OH value, as demonstrated by the presence of gallic acid. Catechins (e.g., catechin and epicatechin) have been observed to stabilize the catechol radical, ensuring a rapid reaction with peroxyl radicals [77].

Zhu et al. [78] documented a strong positive correlation (*R*^2^ = 0.996) between TPC and FRAP values in red wines, which confirmed that phenolic compounds were primarily responsible for reducing Fe^3+^ with the wine antioxidants. Specifically, anthocyanins (*R*^2^ = 0.984), including monoglucoside anthocyanins (*R*^2^ = 0.949), exhibited high correlation coefficients. According to the study by Stratil et al. [27], the correlation coefficient values between TPC and FRAP reached 0.96 and 0.79, for red and white wines, respectively. As calculated in the cited work, these values were slightly lower than the correlations found for the TPC–DPPH measurements (0.97 and 0.82 for the red and white wines, respectively), and considerably lower than those of TPC–ORAC (0.88 and 0.65, respectively). In all cases, red wines exhibited higher antioxidant and antiradical capacities, which the authors attributed to the presence of anthocyanins. The findings of the present study are consistent with these results. Furthermore, our data show that maceration increased the antioxidant/antiradical capacity of the wines, as determined by both colorimetric (FRAP and DPPH) and the fluorimetric ORAC-fl methods, with the greatest increase observed after four days of maceration. Prolonged maceration still favored an upward trend of the parameters monitored with FRAP, although the rate of increase was considerably slower. For DPPH and ORAC-fl tests, the differences between the antioxidant capacity of wines macerated for 4 and 20 days were no longer statistically significant. It should be emphasized that a highly significant effect (*p* < 0.01) of the tested grape cultivar was obtained with all the methods employed. Astonishingly, the DPPH and FRAP tests revealed higher values for ‘Johanniter’ relative to ‘Regent’, whereas ORAC-fl yielded much stronger antiradical scavenging activity for ‘Regent’.

There is only limited data available from other studies on the antioxidant capacities of Polish wines produced from the ‘Johanniter’ and ‘Regent’ berries. Kapusta et al. [21] studied both wine types and, applying the DPPH method, they reported 1.00 and 6.51 mmol/L TE for ‘Johanniter’ and ‘Regent’, respectively. The respective values obtained by these authors with the FRAP assay were 1.50 and 9.05 mmol/L TE. Wojton et al. [22] compared the wines using FRAP and obtained respective values of 3.95 and 25.20 mmol/L TE. In turn, Bednarska et al. [23] documented 3.14 mmol/L TE for ‘Johanniter’ with the DPPH assay, whereas Socha et al. [24] reported 5.38 mmol/L TE with FRAP. It should be emphasized here that maceration time was not specified in the cited studies.

The EPR spectroscopic measurements were performed with the use of two spin labels: water-soluble TEMPO and DPPH dissolved in ethanol (for the method details see Section 4.5.7). The free electron of the TEMPO molecule is delocalized within the N–O bond, providing resonance stabilization and exceptional stability due to its structural features, such as the absence of π bonds and the presence of methyl groups that cover the N–O group. TEMPO does not initiate free radical reaction cascades and only reacts with certain strong reducing agents, such as ascorbic acid, SO_2_, and some flavonols. It acts like a standard redox molecule, reducing antioxidants via the ET-PT mechanism [79,80]. TEMPO has a higher redox potential than DPPH and is less electrophilic; therefore, it reacts with phenols much less readily than DPPH [81].

The EPR studies revealed an initial rapid reduction in TEMPO, followed by a less pronounced gradual reduction, which may be due to a smaller pool of strongly reducing agents in wines. Among the strongest wine reducers were, most likely, glutathione and SO_2_, while the reactions with the phenolic constituents revealed a much slower reducing power and did not reach a plateau within 62 min. In addition, the presence of strongly buffering tartaric acid and its salts resulted in a high acidity of the reaction solution, in which most of the phenols remained undissociated, making the reaction even slower. In contrast, the reaction with DPPH and the antioxidants occurred very rapidly. By 62 min, most of DPPH was reduced; however, the reaction was not completed within the time of observations (see Figure 11). As discussed earlier, with regard to the FRAP and DPPH colorimetric reactions, macerated ‘Johanniter’ wine contained stronger reducing agents than macerated ‘Regent’. Moreover, as shown by kinetic analyses, the J4 reaction with both TEMPO and DPPH proceeded faster than the R4 reaction with these spin labels. The strongest phenolic reducing agents, such as gallic acid or catechins, probably reacted with TEMPO. Our LC–MS analyses confirmed that a lot of gallic acid, caftaric acid (a hydroxycinnamic acid), and epicatechin dimer were extracted into J4 (Figure 2). The latter two compounds are distinguished by the presence of two –OH groups in the -*ortho* position, which makes them highly reactive, both by the ET-PT and HAT mechanisms. Finally, as regards the EPR direct radical measurements, we stress the fact that the available literature data is lacking, especially in relation to the assessment of antiradical compounds in wines.

Summing up, attempts to establish the antioxidant properties of biological samples, such as those of wines, represent a broad field of scientific research, still riddled with simplifications, uncertainties, and even contradictions. Currently, there is no single, universal method for assessing antioxidant potential, and none of the available analyses provides all the necessary information about the tested antioxidant [82]. Common antioxidant testing methods merely quantify antioxidant capacity without considering reactivity and effectiveness over time. Therefore, this work, although insightful and based on varied and systematic analytical approaches, should be regarded as an introductory step only, which requires more in-depth and methodologically advanced studies of antioxidants in macerated wines.

## 4. Materials and Methods

### 4.1. Hybrid Grapes

The ‘Johanniter’ grapevine is a frost-resistant (–24 °C) non-specific hybrid. It was developed in 1968 by Johannes Zimmermann of the Staatliches Weinbauinstitut Freiburg Abteilung Weinbau (State Institute for Wine in Freiburg, Germany), by crossing the cultivar ‘Riesling Weiss’ with the unnamed Freiburg line 589-54 (‘Rulander’ × ‘Gutedel’) [83]. The cultivar manifests notable vitality, particularly in moderately moist sandy loam soils. The onset of fruit ripening under Polish conditions occurs in early October, and the fruits exhibit light coloration. ‘Johanniter’ demonstrates resistance to fungal diseases and moderate resistance to gray mold, requiring minimal protection against powdery mildew. The yield exhibits high stability and productivity. The wines produced from this cultivar have been noted for their delicate aromas of green apple, pear, and peach, making the grapes particularly well-suited for the production of sparkling wines [84]. The area dedicated to ‘Johanniter’ cultivation in Poland was 46.42 hectares in 2023, ranking it sixth among grape cultivar crops in the country [85].

The ‘Regent’ vine is a hybrid cultivar that was obtained upon cross-breeding the ‘Diana’ cultivar (‘Silvaner’ × ‘Miller-Thurgau’) and the ‘Chamburcin’ cultivar (‘Seyve Villard 12-417’ × ‘Chancellor’) in 1967 by Gerhard Alleweldt of the Julius Kühn-Institut (JKI), Bundesforschungsinstitut für Kulturpflanzen, Institut für Rebenzüchtung Geilweilerhof in Germany [83]. The cultivar was registered in Germany in 1996 (Vitis International Cultivar Catalogue, No. 4572). It is composed of the following grape species: *Vitis vinifera* L. (78.5%), *V. rupestris* S. (14.6%), *V. labrusca* L. (3.3%), *V. berlandieri* P. (1.6%), *V. lincecumii* V. (1.1%), and *V. riparia* M. (1%) [12]. The shrub growth is moderately strong. It demonstrates a certain degree of frost resistance, with a threshold of −24 °C, and notable resistance to fungal diseases, a trait that facilitates its cultivation in organic vineyards. The fruit ripening under Polish conditions occurs in late September and October. Berries exhibit low susceptibility to powdery mildew infection, and their coloration ranges from dark to dark blue. The wine produced from this cultivar is of notable quality, exhibiting an intense red color and a moderate tannin content [84]. In 2023, the area dedicated to the cultivation of ‘Regent’ grapes in Poland amounted to 66.47 hectares, ranking third among the country’s grape cultivars in terms of the cultivated area [85].

The fruits of both grape cultivars were derived from Janowice Vineyard (southern Poland; 49°54′13.65″ N; 20°50′20.43″ E), founded in 2013 and approved for official sales with certification. It is located on a southwestern slope at an altitude of 279 m a.s.l., managed in accordance with conventional practices, employing authorized products for plant protection.

### 4.2. Chemicals and Reagents

AAPH (2,2′-azobis(2-methylpropionamidine), DPPH (2,2-diphenyl-1-picrylhydrazyl), DTNB (5-5′-dithiopropionic acid), fluorescein sodium salt, Folin–Ciocalteu reagent, pararosalinine (4,4′-(4-iminocyclohexa-2,5-dienylidenemethylene)dianiline), TEMPO ((2,2,6,6-tetramethylpiperidin-1-yl)oxyl), TPTZ (2,4,6-tris(2-tripyridyl)-s-triazine), and Trolox (6-hydroxy-2,5,7,8-tetramethylchroman-2-carboxylic acid) were obtained from Sigma-Aldrich (Merck Life Science, Darmstadt, Germany). Most of the chromatographic standards were from Sigma-Aldrich (Saint Louis, MO, USA) (catechin, epicatechin, resveratrol, piceid, caftaric acid, caffeic acid, *p*-coumaric acid, gallic acid); procyanidin B2 was from PhytoLab (Vestenbergsgreuth, Germany). L-Malic acid, tartaric acid, formaldehyde, and other salts and compounds were of analytical grade and were purchased either from Chempur (Piekary Śląskie, Poland) or Avantor (Gliwice, Poland). Acetonitrile and formic acid for UHPLC–MS analyses were LC–MS grade (Sigma-Aldrich). Methanol used for EPR measurements was of a HPLC gradient grade (Merck KGaA, Darmstadt, Germany). All aqueous solutions were prepared with deionized water (Christ-Aqua, AG, Aesch, Switzerland).

### 4.3. Preparation of ‘Johanniter’ Wine for Testing

Fruit harvesting was conducted manually on 13 October 2023. Grapes were de-stalked and then crushed by hand, yielding 20 L of pulp, which was then transferred in its whole volume into a single 30 L plastic fermentation tank. The basic parameters of the must, namely sugar extract, pH, and total acidity, were then evaluated. The pulp was sulfurized with potassium pyrosulfate (50 mg/L), treated with the Vitaferment must conditioner (30 mg/L) (Lamothe-Abier, Bordeaux, France).

The starter was inoculated with Zymaflore X16 yeast (Laffort, Bordeaux, France), prepared according to the manufacturer’s instructions, and then thoroughly mixed. The yeast inocula were administered on day 0, that is, immediately after preparation of the must and at the start of the maceration process observation. Next, three liters of pulp were extracted from the settling and subjected to mechanical compression in a manual basket press. The resulting must of 1.85 L volume was transferred into a 2 L glass tank, sealed with a stopper equipped with a fermentation tube, and labeled as “J0”. The fermentation container containing the residual pulp was hermetically sealed with a lid that also featured a fermentation tube and then stored at 24 °C. The onset of fermentation was observed after 33 h. The pulp in the fermentation container was stirred twice a day throughout the fermentation process. After four days of maceration, three liters of fermenting pulp were extracted from the fermentation container and marked as the wine sample J4. The aforementioned steps were repeated on days 8, 12, 16, and 20 of maceration, yielding samples J8, J12, J16, and J20, respectively. On day 30 after yeast inoculation, all samples were decanted into 2 L glass bottles under an argon (99.99% purity) atmosphere, and then the free space in each bottle was filled with Ar. The bottles were sealed with stoppers equipped with fermentation tubes and stored at 15 °C. After another 7 days, cold stabilization was initiated to precipitate the tartaric acid salts. For this purpose, the samples were stored at –5 °C for 14 days, followed by reprecipitation under Ar protection. Finally, a 1.7 L volume of wine was produced from each sample, sulfurized with potassium pyrosulfate (50 mg/L), and the residual volumes in the bottles were filled with Ar. The bottles were then secured with stoppers with fermentation tubes and stored at 10 °C.

### 4.4. Preparation of ‘Regent’ Wine for Testing

Fruit harvest was carried out manually on 6 October 2023. After grape de-stalking and manual crushing, a total of 20 L of pulp was obtained, transferred in its whole into a single plastic fermentation 30 L container, and next, the must’s basic parameters were evaluated as described in Section 2.3. The maceration process (0, 4, 8, 12, 16, and 20 days) was conducted in a way analogous to the treatment of ‘Johanniter’ (Section 4.3), yielding wine samples marked as R0, R4, R8, R12, R16, and R20, respectively. Yeast inoculation of the must, as well as all other steps of processing for this wine, were the same as described above for ‘Johanniter’.

**Figure 12:**
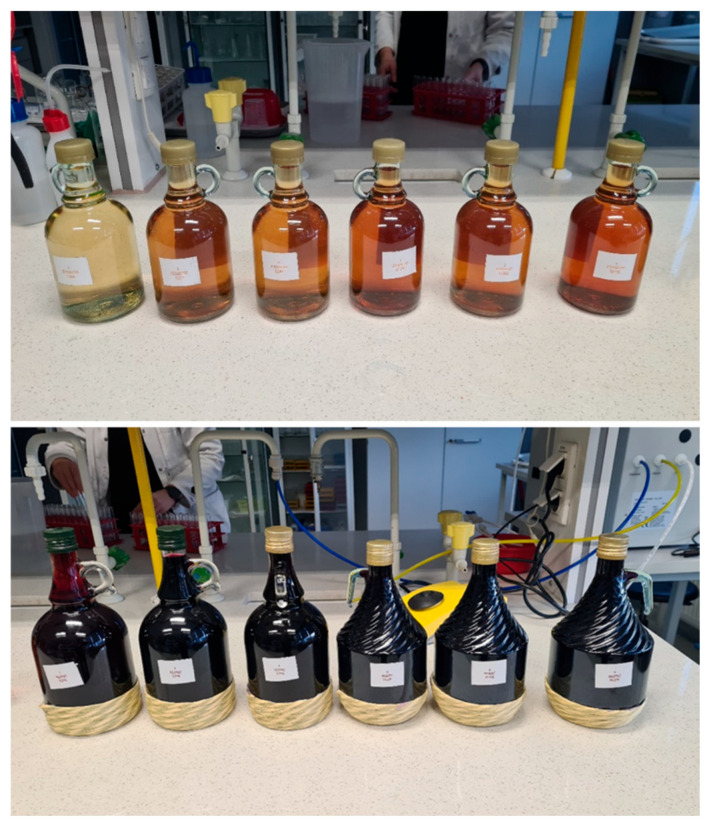
‘Johanniter’ (**top**) and ‘Regent’ (**bottom**) wine samples prepared for analysis. The order of samples from left to right: non-macerated wines followed by wines macerated for 4, 8, 12, 16, and 20 days.

It is noteworthy that for both the ‘Regent’ and ‘Johanniter’ grapes, the vinification process was carried out in a consistent manner under identical conditions. The distinguishing characteristics of ‘Regent’ wine were due to the use of specific yeast strains (Excellence FR, Lamothe-Abier, Bordeaux, France) and Optiflore (30 mg/L) must conditioner (Lamothe-Abier, Bordeaux, France). Prior to the beginning of the study, the bottles were sealed with airtight caps, transported to the University of Agriculture in Krakow (Figure 12), and then stored at 8 °C in the dark until testing. During the course of the tests, the space above the wine surfaces was meticulously protected using an Ar layer, and the bottles were subsequently sealed.

### 4.5. Analytical Methods

#### 4.5.1. Basic Oenological Characteristics of Must and Wine

Oenological parameters of ‘Johanniter’ and ‘Regent’ musts and wines were determined upon triplicate analyses of the samples.

##### Extract Content of Must

The extract content in the musts was measured refractometrically with a hand-held refractometer (Tessa, Renens, Switzerland) and an aerometer at 20 °C.

##### pH of Must and Wine

The pH values of the musts and wine samples were measured at 20 °C using Voltcraft PHT-200 (Conrad Electronics, Dietlikon, Switzerland) and Elmetron CP-505 (Zabrze, Poland) pH-meters, respectively.

##### Total Acidity of Must

The total acidity of the musts was analyzed in accordance with the protocol for “rectified concentrated must”, OIV-MA-F1-05 (Type IV), described in the Compendium of International Methods for Wine and Must Analysis [86]. The analyses were conducted using a specialized titration kit comprising an automatic Schilling burette, a magnetic stirrer, and a Voltcraft PHT-200 pH-meter (Conrad Electronic, Switzerland). Briefly, 10 mL of must and 10 mL of distilled water were transferred to an Erlenmeyer flask, placed on a magnetic stirrer, heated to 20 °C, and titrated with 0.1 M NaOH until a pH of 7.0 was reached. The total acidity of the must, *TA_m_*, was expressed in grams of tartaric acid per 1 L.

##### Total Acidity of Wine

The total acidity of wines was evaluated with a potentiometric method, according to the protocol OIV-MA-AS313-01 (Type I), as outlined in [86]. In summary, 5 mL of each wine sample was transferred to a 100 mL volumetric flask, and the volume was adjusted to 100 mL with distilled water. The resulting solution was transferred to an Erlenmeyer *Erlenmeyer* flask, placed on a magnetic stirrer, heated to 20 °C and titrated with 0.1 M NaOH until a pH of 7.0 was reached. The total acidity of the wine, *TA*_w_, was expressed in grams of tartaric acid per 1 L.

##### Alcohol Content in Wine

Alcoholic strength was determined by volume at 20 °C, based on distilling the beverage after alkalizing it with a calcium hydroxide suspension to prevent volatile acids from escaping. The density of the distillate, measured using a pycnometer, is equal to the quotient of mass over volume [g/cm^3^]. The alcohol content value was then calculated, following tabulated values [86].

##### Residual Sugar Content in Wine

The residual sugar content (glucose + fructose) was determined enzymatically. The assay consisted of catalytic phosphorylation of glucose and fructose by ATP with hexokinase to form glucose-6-phosphate (G6P) and fructose-6-phosphate (F6P). G6P was oxidized to gluconate-6-phosphate by NADP in the presence of G6P dehydrogenase. The produced NADPH was determined spectrophotometrically at 340 nm and its concentration corresponded to the amount of glucose. In turn, F6P underwent conversion to G6P by phosphoglucose isomerase, and then the determination of G6P with the procedure described above allowed for the quantification of fructose [86,87].

##### Free Sulfur Dioxide Concentration in Wine

Free sulfite was determined with a specific color reagent, pararosalinine (4,4′-(4-iminocyclohexa-2,5-dienylidenemethylene)dianiline) and formaldehyde at acidic pH. The amount of pink chromogen was measured spectrophotometrically at 575 nm and was proportional to the sulfite content in the sample [88].

##### Total Sulfur Dioxide Concentration in Wine

In an alkaline environment, sulfites react with 5-5′-dithiobis(2-nitrobenzoic acid) (DTNB). The cleavage of disulfide bonds by DTNB results in the stoichiometric formation of yellow 5-mercapto-2-nitrobenzoate, which absorbs light at a wavelength of 405 nm and is determined colorimetrically [89].

##### L-Malic Acid Content in Wine

L-Malic acid was determined using an enzymatic method involving the oxidation of L-malic acid to oxaloacetate by NAD in a reaction catalyzed by L-malate dehydrogenase. The equilibrium of this reaction typically shifts towards malate. Removing oxaloacetate from the reaction mixture caused the equilibrium to shift towards the formation of oxaloacetate. In the presence of L-glutamate, oxaloacetate was converted to L-aspartate in a reaction catalyzed by glutamate–oxaloacetate transaminase. The produced NADH was measured at 340 nm, and its amount was proportional to the content of L-malate originally present in the sample [86,87].

##### Tartaric Acid Content in Wine

The content of tartaric acid (or tartrate) was measured with a colorimetric method. Under acidic conditions, tartrate reacts with vanadium salts to stoichiometrically form a yellow-orange complex, metapervanadyl tartrate, the amount of which is measured spectrophotometrically at 520 nm [90].

#### 4.5.2. Total Phenolic Content in Wine

The total phenolic content (TPC) of the wines was evaluated using the Folin–Ciocalteu test [91]. The wine samples were diluted with water 300-fold prior to the measurements. A volume of 2.5 mL of each wine sample, 0.25 mL of 25% Na_2_CO_3_, and 0.125 mL of twice-diluted Folin–Ciocalteu reagent were introduced into test tubes and mixed thoroughly by vortexing. The absorbance of the samples containing the wine or gallic acid standard solutions was measured at 760 nm against water (UV/VIS V-530 spectrophotometer, Jasco, Tokyo, Japan). The measurements of wine samples were carried out in triplicate, whereas gallic acid standard solutions were made in duplicate. The concentration of phenolic compounds in wine was then calculated using a standard curve (*y* = 0.3296*x* + 0.0679, *R*^2^ = 0.996), which was prepared based on the absorbance measurements of gallic acid solutions. The results were expressed in grams of gallic acid equivalents, GAE, per 1 L of wine.

#### 4.5.3. Phenolic Profiling of Wine with UHPLC–MS

Phenolic profiling of the wines was carried out employing Ultra-High-Performance Liquid Chromatography (UHPLC) coupled with mass spectrometry (MS). The analyses were performed using the samples J0, J4, J20, R0, R4, and R20. In addition, due to our particular interest in ‘Johanniter’ wines, and their specific features (see Section 3), we also examined the sample J16. Prior to the measurements, the wines were filtered (Thomson SINGLE STEP, 0.2 µm PTFE filter vials; Thomson Instrument Company, Oceanside, CA, USA). Next, they were analyzed using a Thermo Ultimate 3000RS (Thermo Fisher Scientific, Waltham, MA, USA) UHPLC system, equipped with a photodiode array detector (PDA), and a charged aerosol detector (CAD). The system was hyphenated with a Bruker Impact II Q-TOF mass spectrometer (Bruker Daltonics GmbH, Bremen, Germany) fitted with an electrospray ion source. The samples were chromatographed on an ACQUITY UPLC HSS T3 column (2.1 × 100 mm, 1.8 µm; Waters, Milford, MA, USA), at 45 °C. The mobile phase consisted of solvent A (0.2% formic acid in Milli-Q water) and solvent B (0.2% formic acid in acetonitrile). The elution method started from a 1 min isocratic segment (2% B), followed by a 26 min concave gradient (2–40% B; Chromeleon gradient No. 7). The column was subsequently washed with 99% B (1 min), and the mobile phase returned to the initial conditions (2% B); the total time of analysis was 32 min. The flow rate was 400 µL min^–1^, and the injection volume was 5 µL. The mass spectrometer operated in positive and negative ion modes (in separate LC–MS analyses). The scanning range was from *m*/*z* 80 to *m*/*z* 1500. The negative mode settings: capillary voltage 3000 V; dry gas (nitrogen) temperature 200 °C, dry gas flow 6 L min^−1^; nebulizer pressure 0.7 bar; collision RF 700 Vpp; transfer time 90 µs; prepulse storage time 10 µs. Collision energy (collision gas: argon) was set automatically in the range from 7 to 105 eV, depending on the *m*/*z* values of fragmented ions. The positive mode settings: capillary voltage 4500 V; dry gas (nitrogen) temperature 200 °C, dry gas flow 6 L min^–1^; nebulizer pressure 0.7 bar; collision RF 700 Vpp; transfer time 87.5 µs; prepulse storage time 10 µs. Collision energy was set automatically in the range from 9 to 50 eV, depending on the *m*/*z* values of fragmented ions. The acquired spectra were calibrated at the beginning of each LC–MS analysis using sodium formate clusters introduced to the ion source through a 20 μL loop. Most of phenolics and other aromatic compounds were tentatively identified on the basis of their high-resolution mass spectra (employing the following analytical tools: a database search program Compound Crawler 3.3 (Bruker, Billerica, MA, USA), SIRIUS (version 5.8.3) [92], and MS Dial (RIKEN, version 5.4.241021) [93] open source tools for the analysis of LC–MS data, as well as the MS-DIAL metabolomics MSP spectral kit, containing publicly available ESI-MS/MS data obtained from authentic standards (SysteMSOmics lab), UV spectra, and the literature. In addition, gallic acid, caftaric acid, caffeic acid, *p*-coumaric acid, catechin, epicatechin, and procyanidin B2 were unambiguously identified using authentic standards.

#### 4.5.4. Antioxidant Capacity of Wine

The antioxidant capacity of the wines was measured using the Ferric ion Reduction Antioxidant Power (FRAP) assay [94]. The FRAP working solution was prepared prior to use by combining 30 mL of 0.001 M TPTZ solution in 96% ethanol, 30 mL of 0.02 M FeCl_3_ solution, and 300 mL of 0.3 M acetate buffer. Subsequently, 3 mL of the FRAP working solution was added to the test tubes containing wine samples diluted 100-fold with water or with Trolox standards. Thereafter, the mixtures were vortexed and incubated for 15 min at RT in the dark. Absorbance measurements were performed at 595 nm against water (UV/VIS V-530 Spectrophotometer, Jasco, Japan). The measurements of wine samples were conducted in triplicate, while Trolox standard solutions were carried out in duplicates. After data acquisition, a standard curve (*y* = 0.4053*x* + 0.1477; *R*^2^ = 0.917) was generated, and the antioxidant capacity results were expressed in mmol of Trolox equivalents, TE, per 100 mL of wine.

#### 4.5.5. DPPH Antiradical Capacity of Wine

The antiradical capacity was evaluated using the DPPH radical colorimetric assay [95]. Briefly, 2.8 mL of a 0.1 mM DPPH solution in 96% ethanol was added to the test tubes containing 0.2 mL either wine samples or Trolox standard solutions. The ‘Johanniter’ and ‘Regent’ wines were diluted 10 and 5 times, respectively. The mixtures were vortexed and subsequently incubated for 15 min at room temperature (RT) in the dark. The absorbance measurements were carried out at 517 nm against the blank containing the appropriate wine sample dilution using a UV/VIS V-530 spectrophotometer (Jasco, Japan). Measurements of the wine samples were performed in triplicate, whereas those of the Trolox were conducted in duplicate. Following data acquisition, a standard curve (*y* = –1.3508*x* + 1.0456; *R*^2^ = 0.998) was generated using the Trolox standard solutions. The antiradical capacity results were expressed in mmol of Trolox equivalents, TE, per 100 mL of wine.

#### 4.5.6. Peroxyl Antiradical Capacity of Wine

The ORAC-fl (Oxygen Radical Absorbance Capacity) assay is a standardized fluorescein-based measurement method for determining the antiradical capacity of a substance [74]. The measurements were performed in triplicate, following the procedures described by Ou et al. [96] with modifications. Briefly, a fluorescein 4 µM stock solution in a 100 mM phosphate buffer, pH 7.4, was diluted 80-fold to obtain a 50 nM working solution and kept at RT in the dark. An aqueous solution of AAPH (153 mM) was stored at –80 °C, then thawed and warmed to RT immediately before analysis. Standard aqueous solutions of Trolox were prepared at concentrations of 3.125 mM, 6.25 mM, 12.5 mM, 25 mM, 50 mM, and 75 mM. Wine samples were diluted with redistilled water immediately before measurements; the dilution rates were 800 or 1000-fold for the ‘Johanniter’ wine and 1000 or 1200-fold for ‘Regent’. Fluorescein emission was observed using a HITACHI F4500 spectrofluorimeter (Hitachi High-Tech, Tokyo, Japan), operating under a kinetic mode (“time scan”) with a measurement time of 1500 s. The specimens, each of 2 mL in volume, were placed in a thermostated (37 ± 0.1 °C) quartz cuvette with a 1 cm optical path and mixed with a magnetic stirrer. The excitation wavelength was set at λ_exc_ = 485 nm, and the emission at λ_em_ = 515 nm, with monochromator slit widths of 5 nm. As a reference sample, a fluorescein working solution in a phosphate buffer was used to adjust the system to the maximum fluorescence signal as well as to check for fluorescein photostability. To measure the antiradical activity of the tested wines, the samples were prepared by mixing 1.5 mL of fluorescein solution and 0.25 mL of appropriately diluted wine. Following a 3 min temperature stabilization, 0.25 mL of AAPH solution was added, which initiated fluorescence signal recording. The “ORAC-blank” sample was measured to monitor the kinetics of fluorescein quenching by free radicals generated by AAPH in the absence of antioxidants. A standard curve (*y* = 5243.3*x* + 61981; *R*^2^ = 0.982) was generated by analyzing the consecutive Trolox solutions added instead of wine samples. The antiradical capacities of wines (ORAC values) were calculated as described by Cao and Prior [75] employing the Hitachi F4500 FL Solutions Program No. 2500607-10 (Hitachi High-Tech, Tokyo, Japan). The ORAC values of wine samples were interpolated based on the Trolox standard curve, and the results were expressed as mmol of Trolox equivalent, TE, per 100 mL of each tested wine.

#### 4.5.7. Nitroxyl and Hydrazyl Antiradical Capacity of Wine

The analyses were performed using a custom-built low-frequency (L-band, 1.2 GHz) electron paramagnetic resonance (EPR) spectroscopy [97]. The tested parameter was the ability of antioxidant compounds to reduce the synthetic stable radicals: DPPH dissolved in 100% methanol, and TEMPO dissolved in water. The calculated amplitude of the central peak of the EPR spectrum is proportional to the total radical content. A custom-built spectrometer (University of Agriculture in Krakow, Poland) operated under the following settings: maximum microwave power of 16 mW, magnetic modulation amplitude: 2 Gauss at 34 kHz, sweep range: 35 Gauss, time constant: 10 ms, sweep time: 20 s, break time: 5 s. The J0, J4, J20, R0, R4, and R20 sample volumes (0.35 mL of wine + 0.35 mL of either DPPH or TEMPO solutions) were placed in 2 mL Eppendorf tubes inside a surface-loop resonator. The amplitudes were normalized to the independently recorded signal of a blank sample (0.35 mL of 10% ethanol + 0.35 mL of DPPH or 0.35 mL of water + 0.35 mL of TEMPO) and expressed as a percentage of a signal reduction. The data for each run was obtained by averaging five individual scans, with starting times of 0, 5, 10, 15, 20, 30, 40, and 60 min, and ending times of 2, 7, 12, 17, 22, 32, 42, and 62 min. Each sample was measured in three replicates. The resulting EPR data were subsequently analyzed using the unique internal computer software module developed by the spectrometer constructors [97].

### 4.6. Statistical Analysis

Statistical analyses of Section 4.5.2, Section 4.5.3, Section 4.5.4, Section 4.5.5 and Section 4.5.6 were conducted using the STATISTICA 13 software. The results were subjected to a two-factor analysis of variance (ANOVA), with factors relating to the type of wine and maceration time. This was followed by a Tukey post hoc test, assuming a significance threshold of *p* < 0.05 or *p* < 0.01 to make a point of highly significant differences between sample parameters. Statistically significant differences between individual mean values were denoted by different letters.

For phenolic multivariate analysis, feature tables containing peak heights of identified compounds were prepared using MS Dial v. 5.4.241021. Data obtained in positive ion mode were used in the case of red wines, while negative ionization data were used for white wines. Hierarchical clustering analysis was performed using BiostatFlow v. 2.9.6 webtool (http://biostatflow.org/).

## 5. Conclusions

Wines obtained from hybrid grapes, white ‘Johanniter’ and red ‘Regent’, grown under the temperate climate of central Europe, require maceration to become enriched in total phenols as well as to increase their antioxidant and antiradical properties. Our work reveals that the tested hybrid grapevine cultivars produce fruits with distinct phenolic profile characteristics, differing from that of macerated *Vitis vinifera* wines. For the first time, systematic studies were performed regarding the influence of maceration time on the content and activity of bioactive compounds in ‘Johanniter’ and ‘Regent’ wines. A maceration period of only 4 days was found to be critical for must beneficiation with health-promoting compounds. Maceration of white grapes, a relatively rare treatment, in the case of the ‘Johanniter’ cultivar, enabled the production of a beverage with an amber hue, reminiscent of orange wine, which exhibited antioxidant and antiradical potential similar to or even exceeding that found for the red wine. Extensive experimenting that employed varying methodologies allowed for the assessment of the health-beneficial properties of the wine through the application of analytical techniques based on different molecular mechanisms. In particular, we applied two methods very rarely used to characterize musts and wines, namely electron paramagnetic resonance spectroscopy (EPR) and ORAC fluorimetric assay. The first provided valuable information on the specific and direct scavenging activity against nitroxyl or hydrazyl radicals, and the second one enabled the monitoring of the reduction capacity of peroxyl radicals.

The collected data clearly show that hybrid grapevine cultivars grown on the slopes of southern Poland, one of the coldest wine-growing regions in the EU, may yield fully balanced and nutritious wines. Their characteristics reveal high phenolic content and similar antioxidant capacities compared to wines produced in regions with a rich, centuries-old winemaking tradition. The obtained results are therefore promising in the context of progressive climate change, increasing consumers’ demand for products containing bioactive compounds, as well as in terms of promoting viticulture in Poland and other countries of the region. Our study also justifies the need to optimize the production of wines and, more specifically, the need for comprehensive research on the phenolic content and composition of white wines.

## Figures and Tables

**Table 1:** Basic parameters of the ‘Johanniter’ and ‘Regent’ grape musts.

Cultivar	Extract (°Brix)	pH	Total Acidity, *TA*_m_ (Tartaric Acid eq., TAE; g/L)
‘Johanniter’	20	3.26	7.12
‘Regent’	18	3.62	8.33

**Table 2:** Basic oenological parameters of the ‘Johanniter’, J, and ‘Regent’, R, wines, subjected to 4-, 8-, 12-, 16-, or 20-day maceration. J0, R0—unmacerated wines.

Wine Sample	pH	Total Acidity *TA*_w_ (TAE; g/L)	Alc. Vol. (%)	Residual Sugar (g/L)	SO_2_ Free (mg/L)	SO_2_ Total (mg/L)	L-Malic Acid (g/L)	Tartaric Acid (g/L)
J0	3.23	8.69	12.0	0.08	<3 *	66	2.3	2.1
J4	3.59	8.53	11.4	0.08	5	40	2.4	1.7
J8	3.65	8.54	11.5	0.05	13	37	2.3	1.6
J12	3.63	8.10	11.7	0.06	<3 *	15	2.3	1.6
J16	3.65	8.06	11.5	0.05	3	21	2.1	1.5
J20	3.67	7.93	11.6	0.05	<3 *	35	2.3	1.4
R0	3.75	8.19	10.0	0.03	<3 *	29	1.70	2.3
R4	3.98	7.24	9.5	0.10	3	14	0.03	2.7
R8	3.94	6.96	9.5	0.09	6	<10	0.07	2.4
R12	3.91	6.99	9.4	0.09	13	19	0.04	2.4
R16	3.87	7.20	9.6	0.06	9	21	0.03	2.2
R20	3.86	8.08	9.3	0.09	12	19	0.04	2.3

Data marked with an asterisk represent values below the limit of detection (LOD = 3 mg/L) in analyses of free SO_2_.

## Data Availability

Data are contained within the article and Supplementary Materials. Additional raw data are available from the authors upon request.

## Supplementary Materials

The following supporting information can be downloaded at: https://www.mdpi.com/article/10.3390/molecules31010179/s1, Table S1. Preliminary sensory evaluation of wines produced from ‘Johanniter’ and ‘Regent’ grape cultivars, showing main changes in color, aroma, and taste upon the maceration process (carried out according to [98]); Table S2. Total phenolic content, TPC, in the ‘Johanniter’ and ‘Regent’ wines determined by the Folin–Ciocalteu assay; Table S3. Tentative identification of phenolics and other aromatic compounds in the ‘Johanniter’ wines (identification level according to [99]); Table S4. Tentative identification of phenolics and other aromatic compounds in the ‘Regent’ wines (identification level according to [99]); Table S5. Antioxidant capacities of the ‘Johanniter’ and ‘Regent’ wines determined by the FRAP assay; Table S6. Antiradical capacities of the ‘Johanniter’ and ‘Regent’ wines determined by the DPPH colorimetric assay; Table S7. Antiradical capacities of the ‘Johanniter’ and ‘Regent’ wines determined by the ORAC-fl assay.

## Abbreviations

The following abbreviations are used in this manuscript:

AAPH	2,2′-azobis(2-methylpropionamidine)
BDE	bond dissociation energy/enthalpy
DPPH	2,2-diphenyl-1-picrylhydrazyl
DTNB	5,5′-dithiobis(2-nitrobenzoic acid)
ET	electron transfer
EPR	Electron Paramagnetic Resonance
F6P	fructose-6-phosphate
FRAP	Ferric ion Reduction Antioxidant Power
G6P	glucose-6-phosphate
GAE	gallic acid equivalent
HAT	hydrogen atom transfer
J0,4…	‘Johanniter’ wines obtained after appropriate maceration time, in days
KHT	potassium hydrogen tartrate
MLF	malolactic fermentation
LOD	limit of detection
LC–MS	Liquid Chromatography–Mass Spectrometry
ORAC-fl	fluorescein-based Oxygen Radical Absorbance Capacity
PCET	proton-coupled electron transfer
PT	proton transfer
PhO–	phenolate ion
R0,4…	‘Regent’ wines obtained after appropriate maceration time, in days
ROO•	peroxyl radical
RT	room temperature
SET	single electron transfer
SPLET	sequential proton loss electron transfer
TAE	tartaric acid equivalent
*TA*_m_	total acidity of must
*TA*_w_	total acidity of wine
TE	Trolox equivalent
TEMPO	2,2,6,6-tetramethylpiperidine-1-oxyl
TPC	total phenolic content
UHPLC	Ultra-High-Performance Liquid Chromatography
