# Supplementary material for: Optimizing Wine Production from Hybrid Cultivars: Impact of Grape Maceration Time on the Content of Bioactive Compounds
Source: Molecules. 2026 Jan 3;31(1):179. doi: 10.3390/molecules31010179 (PMC12787621; doi:10.3390/molecules31010179)
Supplement: Supplementary file 1 [file molecules-31-00179-s001.zip › molecules-4046690-supplementary.pdf]

Table S1. Preliminary sensory evaluation of wines produced from 'Johanniter' and 'Regent' grape cultivars, showing main changes in color, aroma, and taste upon the maceration process.

			**'Johanniter'**	**'Regent'**
Appearance		Intensity	medium → deep	deep
		Color	gold → amber	garnet → tawny
Nose		Intensity	light → medium	pronounced
		Aroma characteristics	primary/tertiary	primary/tertiary
Palate		Sweetness	dry	medium → off-dry → dry
		Acidity	high	medium → high
		Tannin	medium → high	medium → high
		Alcohol	medium	low → medium
		Body	medium → full	medium → full
		Flavor intensity	light medium → medium	medium → pronounced
		Flavor characteristic	primary/tertiary	primary/tertiary
		Finish	short → medium	medium
Describing aroma and flavor	Primary aromas and flavors	Fruit	gooseberry, grape, apricot, nectarine	gooseberry, cherry, blackcurrant, blackberry
		Herbaceous	asparagus	tomato leaf
		Spice	white pepper	–
	Tertiary aromas and flavors		hazelnut → ginger	wet leaves

The table was composed according to: Wine & Spirit Education Trust (WSET). *Level 2 Award in Wines: Looking Behind the Label*, Issue 2; Wine & Spirit Education Trust: London, UK, 2019

Table S2. Total phenolic content, TPC, in the 'Johanniter' and 'Regent' wines determined by the Folin-Ciocalteu assay.

**Maceration time** (days)	**Total phenolic content, TPC** (GAE; g/L)	
	**'Johanniter'**	**'Regent'**
**0**	**0.64 ± 0.0 a**	**0.93 ± 0.02 b**
4	2.77 ± 0.11 c	2.98 ± 0.03 c
8	2.89 ± 0.14 c	3.36 ± 0.03 d
12	2.99 ± 0.02 c	3.87 ± 0.21 e
16	3.27 ± 0.11 d	3.46 ± 0.03 d
20	3.51 ± 0.01 d	3.77 ± 0.04 e
Average	2.68 ± 0.97 A	3.06 ± 1.03 B

Values marked with different letters differ significantly in a Tukey's post hoc test, assuming a significance threshold $p < 0.01$ to indicate highly significant differences between sample parameters. The lowercase letters (a–e) indicate differences across all data; capital letters (A, B) indicate significance of differences for average values. Data are presented as mean values ± SD; n = 3.

Table S3. Tentative identification of phenolics and other aromatic compounds in the 'Johanniter' wines.

No.	t_R	Ion type	*m/z*	Major fragment ions	Error [ppm]	mσ	Formula	Tentative identification	Identification level*
1	2.14	$[M-H]^-$	169.0143	169.0143 (100), 125.0242 (61)	-0.4	4.9	$C_7H_6O_5$	gallic acid*	1
2	2.43	$[M-H]^-$	609.1264	441.0827 (35), 423.0722 (100), 305.0675 (86), 255.0311 (15), 177.0195 (32), 125.0253 (11)	-2.3	13.9	$C_{30}H_{26}O_{14}$	(epi)GC-(epi)GC	3
3	2.5	$[M-H]^-$	493.1203	331.0678 (100), 169.0145 (30), 125.0260 (6)	-0.7	20.3	$C_{19}H_{26}O_{15}$	gallic acid-Hex-Hex	3
4	3.49	$[M+H]^+$	166.0861	166.0858 (64), 131.0658 (4), 120.0802 (100)	1.2	0.3	$C_9H_{11}NO_2$	phenylalanine	2
5	4.44	$[M-H]^-$	305.0668	305.0672 (100), 261.0770 (9), 219.0668 (10), 179.0356 (5), 167.0348 (6), 125.0244 (9)	-0.5	18.1	$C_{15}H_{14}O_7$	(epi)gallocatechin	2
6	4.49	$[M-H]^-$	593.1307	593.1316 (29), 467.0986 (15), 441.0831 (6), 425.0883 (51), 407.0778 (82), 303.0512 (14), 289.0723 (100), 177.0196 (47), 125.0246 (14)	-1.1	7.1	$C_{30}H_{26}O_{13}$	(epi)GC-(epi)C	3
7	4.65	$[M-H]^-$	206.0462	162.0550 (100), 144.0446 (100), 134.0601 (36)	-1.7	4.3	$C_{10}H_9NO_4$	unidentified aromatic compound	4
8	4.97	$[M-H]^-$	473.0931	473.0948 (16), 341.0886 (100), 311.0620 (6), 179.0355 (60), 135.0440 (28)	1.2	16.1	$C_{19}H_{22}O_{14}$	caffeic acid-tartaric acid-Hex	3
9	5.19	$[M-H]^-$	311.0411	311.0410 (6), 179.0358 (32), 149.0083 (100), 135.0438 (17)	-0.8	1.1	$C_{13}H_{12}O_9$	caftaric acid isomer	3
10	5.24	$[M-H]^-$	457.0983	457.0980 (35), 325.0923 (93), 163.0385 (100), 119.0488 (49)	1	11.7	$C_{19}H_{21}O_{13}$	coumaric acid-tartaric acid-Hex	3
11	5.34	$[M-H]^-$	593.1307	593.1316 (29), 467.0975 (37), 441.0796 (14), 423.0720 (100), 407.0762 (13), 305.0665 (78), 289.0724 (53), 177.0205 (28), 125.0234 (31)	-1.1	20.0	$C_{30}H_{26}O_{13}$	(epi)GC-(epi)C	3
12	5.44	$[M-H]^-$	382.1007	266.0896 (11), 250.0590 (8), 206.0684 (32), 162.0782 (6), 134.0461 (31)	-0.8	3.9	$C_{14}H_{17}N_5O_8$	succinyladenosine / isomer	2
13	5.55	$[M-H]^-$	315.1091	315.1092 (100), 153.0549 (42), 123.0455 (29)	-1.7	3.6	$C_{14}H_{20}O_8$	hydroxytyrosol-Hex / isomer	3
14	5.79	$[M-H]^-$	311.0409	179.0352 (100), 149.0080 (18), 135.0440 (46)	-0.2	3.5	$C_{13}H_{12}O_9$	caftaric acid	1
15	6.02	$[M-H]^-$	181.0506	181.0512 (100), 163.0396 (29), 135.0438 (24), 119.0495 (12)	-0.7	25.9	$C_9H_{10}O_4$	hydroxyphenyllactic acid / isomer	2
16	6.04	$[M-H]^-$	341.0884	237.0597 (2), 181.0512 (4), 179.0340 (3), 163.0385 (3), 161.0443 (2), 135.0431 (2), 107.0491 (6)	-1.7	6.9	$C_{15}H_{18}O_9$	caffeic acid-Hex (?)	3
17	6.11	$[M-H]^-$	616.1091	484.1040 (14), 466.0933 (16), 440.1140 (19), 343.0131 (2),	-0.1	12.0	$C_{23}H_{27}N_3O_{15}S$	caftaric acid-glutathione	2

				272.0891 (100), 254.0785 (11), 211.0072 (25), 192.9967 (12), 167.0173 (24), 149.0083 (13)					
		[M+H]+	618.1240	618.1235 (100), 543.0919 (17), 489.0808 (11), 393.0753 (6), 386.0543 (12), 322.0384 (9), 264.0325 (25), 211.0713 (9), 195.0111 (10), 145.0606 (23)	-0.6	14.3			
18	6.23	[M-H]-	451.1251	451.1255 (14), 289.0717 (100), 245.0822(10)	-1.2	13.7	$C_{21}H_{24}O_{11}$	(epi)catechin-Hex / isomer	3
19	6.29	[M-H]-	353.0880	353.0881 (56), 191.0562 (100), 179.0349 (34), 135.0432 (18)	-0.6	23.2	$C_{16}H_{18}O_9$	chlorogenic acid	3
20	6.96	[M-H]-	593.1302	593.1304 (27), 467.0987 (14), 425.0886 (70), 407.0789 (97), 289.0727 (100), 255.0298 (19), 177.0191 (58)	-0.3	15.2	$C_{30}H_{26}O_{13}$	(epi)C-(epi)GC	3
21	7.11	[M-H]-	487.1106	487.1109 (9), 355.1040 (87), 193.0511 (100), 178.0277 (43), 149.0606 (2), 134.0360 (27)	-2.6	12.6	$C_{20}H_{23}O_{14}$	ferulic acid-tartaric acid-Hex / isomer	3
22	7.25	[M-H]-	897.1884	729.1470 (20), 711.1356 (47), 593.1312 (33), 543.0948 (30), 467.0971 (32), 425.0879 (39), 407.0777 (54), 303.0515 (63), 289.0717 (61), 243.0301 (80), 177.0196 (100)	0.0	18.3	$C_{45}H_{38}O_{20}$	(epi)C-(epi)GC-(epi)GC	3
23	7.92	[M-H]-	295.0462	295.0458 (7), 163.0398 (100), 149.0082 (73), 119.0496 (54)	-1.0	7.7	$C_{13}H_{12}O_8$	coutaric acid 1	3
24	8.06	[M-H]-	881.1949	695.1399 (64), 577.1365 (58), 451.1038 (47), 425.0881 (45), 407.0769 (60), 303.0519 (67), 289.0724 (89), 243.0302 (100), 177.0192 (91)	-1.6	7.0	$C_{45}H_{38}O_{19}$	(epi)C-(epi)C-(epi)GC	3
25	8.10	[M-H]-	295.0465	295.0468 (16), 163.0400 (100), 119.0502 (34)	-1.8	2.5	$C_{13}H_{12}O_8$	coutaric acid 2	3
26	8.26	[M-H]-	577.1361	577.1350 (20), 451.1041 (20), 425.0880 (36), 407.0774 (71), 289.0717 (100), 125.0242 (10)	-1.6	8.9	$C_{30}H_{26}O_{12}$	(epi)C-(epi)C	3
27	8.34	[M-H]-	305.0676	305.0675 (100), 261.0781 (16), 219.0644 (12), 125.0243 (14)	-3.0	10.5	$C_{15}H_{14}O_7$	(epi)gallocatechin	2
28	8.48	[M-H]-	577.1354	451.1043 (23), 425.0874 (42), 407.0780 (67), 289.0727 (100), 255.0305 (11), 125.0259 (10)	-0.4	4.4	$C_{30}H_{26}O_{12}$	(epi)C-(epi)C	3
29	8.79	[M-H]-	289.0721	289.0722 (100), 245.0823 (19)	-1.2	13.3	$C_{15}H_{14}O_6$	catechin	1
30	9.16	[M-H]-	685.1230	685.1241 (35), 603.1512 (45), 451.1035 (100), 433.0929 (52), 289.0716 (31), 281.0459 (24), 151.0385 (9)	0.4	11.5	$C_{32}H_{30}O_{15}S$	(epi)C-ethyl-(epi)C-SO_3H / isomer	3
31	9.44	[M-H]-	179.0351	179.0355 (100), 135.0441 (91)	-0.6	2.0	$C_9H_8O_4$	*trans*-caffeic acid	1
32	9.48	[M-H]-	881.1919	881.1943 (27), 593.1309 (61), 425.0864 (79), 407.0751 (89), 289.0713 (98), 177.0184 (100)	1.7	29.5	$C_{45}H_{38}O_{19}$	(epi)C-(epi)C-(epi)GC	3
33	9.66	[M-H]-	325.0926	325.0932 (27), 163.0394145.0274 (27), (100), 119.0492 (89)	1.0	12.2	$C_{15}H_{18}O_8$	coumaric acid-Hex	3
34	9.74	[M+H]+	231.1130	231.1131 (100), 214.0867 (56), 188.0710 (23), 168.0812 (10), 158.0968 (65)	-0.9	4.8	$C_{13}H_{14}N_2O_2$	tetrahydroharman-3-carboxylic acid / isomer	3

35	10.25	$[M-H]^-$	325.0560	193.0501 (100), 149.0079 (3), 134.0355 (29)	1.5	9.2	$C_{14}H_{14}O_9$	fertaric acid	3
36	10.31	$[M-H]^-$	865.1998	713.1498 (25), 695.1448 (13), 577.1362 (32), 575.1194 (29), 543.0940 (19), 451.1047 (46), 425.0882 (57), 407.0776 (61), 287.0568 (100), 243.0298 (34), 125.0239 (28)	-1.4	7.6	$C_{45}H_{38}O_{18}$	(epi)C-(epi)C-(epi)C	3
37	10.54	$[M-H]^-$	577.1347	451.1037 (16), 425.0892 (26), 407.0784 (48), 289.0725 (100), 125.0237 (10)	0.7	17.5	$C_{30}H_{26}O_{12}$	(epi)C-(epi)C	3
38	10.8	$[M-H]^-$	451.1239	451.1234 (3), 289.0716 (100), 245.0808 (11)	1.6	14.3	$C_{21}H_{24}O_{12}$	(epi)catechin-Hex / isomer	3
39	11.43	$[M-H]^-$	577.1345	577.1345 (17), 451.1034(22), 425.0877 (45), 407.0771 (80), 289.0715 (100), 125.0240 (11)	1.2	8.7	$C_{30}H_{26}O_{12}$	procyanidin B2	1
40	12.22	$[M-H]^-$	685.1221	603.1500 (30), 451.1036 (100), 433.0922 (51), 313.0710 (9), 289.0709 (22), 281.0455 (26), 151.0393 96)	1.7	16.7	$C_{32}H_{30}O_{15}S$	(epi)C-ethyl-(epi)C-SO_3H / isomer	3
41	12.51	$[M-H]^-$	289.0721	289.0726 (100), 245.0825 (22), 221.0822 (7)	-1.1	4.5	$C_{15}H_{14}O_6$	epicatechin	1
42	12.68	$[M-H]^-$	449.1087	449.1088 (100), 287.0558 (39), 269.0452 (53), 259.0610 (45), 125.0239 (11)	0.5	8.8	$C_{21}H_{22}O_{11}$	dihydrokaempferol-Hex / isomer	3
43	12.99	$[M-H]^-$	163.0404	163.0395 (65), 119.0497 (100)	-1.8	8.1	$C_9H_8O_3$	*trans*-*p*-coumaric acid	1
44	13.06	$[M-2H]^{2-}$	576.1280	449.0871 (13), 407.0772 (19), 289.0714 (100)	-1.2	60.3	$C_{60}H_{50}O_{24}$	(epi)C-(epi)C-(epi)C-(epi)C	3
45	13.16	$[M-H]^-$	685.1224	685.1232 (25), 603.1506 (34), 451.1034 (100), 433.0924 (40), 313.0724 96), 289.0718 (17), 281.0455 (21), 151.0382 (5)	1.3	17.9	$C_{32}H_{30}O_{15}S$	(epi)C-ethyl-(epi)C-SO_3H / isomer	3
46	13.16	$[M-H]^-$	881.1939	755.1672 (18), 695.1411 (69), 591.1145 (36), 577.1364 (48), 543.0923 (35), 465.0848 (35), 451.1036 (54), 425.0887 (55), 407.0887 (66), 303.0508 (58), 289.0715 (70), 287.0560 (58), 243.0296 (100)	-0.5	41.8	$C_{45}H_{38}O_{19}$	(epi)C-(epi)C-(epi)GC	3
47	13.37	$[M-H]^-$	197.0453	197.0492 (100), 169.0138 (15), 124.0159 (22)	1.5	7.4	$C_9H_{10}O_5$	ethyl gallate / isomer	3
48	13.70	$[M-H]^-$	366.1192	366.1194 (100), 204.0662 (11), 186.0556 (13), 158.0603 (3), 142.0649 (13)	0.6	3.7	$C_{17}H_{21}NO_8$	indolyllactic acid-Hex / isomer	3
49	14.32	$[M-H]^-$	865.1971	713.1518 (26), 695.1399 (27), 577.1343 (56), 575.1201 (36), 451.1038 (35), 425.0882 (59), 407.0772 (81), 289.0721 (80), 287.0566 (100), 243.0296 (46), 125.0247 (27)	1.7	17.4	$C_{45}H_{38}O_{18}$	(epi)C-(epi)C-(epi)C	3
50	14.64	$[M-H]^-$	865.1971	713.1518 (26), 595.1399 (27), 577.1343, 575.1201 (36), 543.0946 (20), 451.1038 (35), 425.0882 (59), 407.0772 (81), 287.0566 (100),243.0296 (46), 125.0247 (27)	1.7	17.4	$C_{45}H_{38}O_{18}$	(epi)C-(epi)C-(epi)C	3
51	15.46	$[M-H]^-$	204.0664	204.0668 (100), 186.0572 (9), 158.0605 (13), 142.0650 (14), 128.0500 (4)	1.2	1.2	$C_{11}H_{11}NO_3$	indolyllactic acid / isomer	2

52	16.12	[M+H]+	627.1552	465.1020 (46), 303.0501 (100)	0.7	19.1	$C_{27}H_{30}O_{17}$	quercetin-Hex-Hex	3
53	17.29	[M-H]-	389.1239	227.0728 (100), 185.0613 (21)	0.8	14.2	$C_{20}H_{22}O_8$	piceid	1
54	17.34	[M-H]-	605.1686	453.1203 (10), 315.0880 (100), 289.0728 (96), 245.0817 (10), 151.0393 (5)	-3.6	19.0	$C_{32}H_{30}O_{12}$	(epi)C-ethyl-(epi)C	3
		[M+H]+	607.1811	455.1351 (2), 317.1023 (100)291.0863 911), 165.0547 (12)	-0.2	25.3			
55	17.71	[M-H]-	893.2311	603.1528 (49), 577.1379 (45), 451.1056 (74), 411.0929 (42), 425.0878 (50), 407.0790 (31), 315.0876 (57), 289.0720 (100)	-1.4	40.0	$C_{47}H_{42}O_{18}$	(epi)C-(epi)C-ethyl-(epi)C	3
56	18.83	[M-H]-	477.0675	477.0669 (17), 301.0349 (100), 255.0288 (10)	0.0	16.2	$C_{21}H_{18}O_{13}$	quercetin-HexA	3
		[M+H]+	479.0820	479.0814 (12), 303.0501 (100)	0.1	9.2			
57	18.99	[M-H]-	449.1088	449.1106 (89), 303.0517 (27), 287.1507 (100), 285.0413 (57), 227.1292 (16), 151.0037 (17)	2.3	23.4	$C_{21}H_{22}O_{11}$	taxifolin-dHex / isomer	3
		[M+H]+	451.1230	451.1236 (7), 305.0659 (100), 287.0554 (14), 259.0602 (18)	1.1	9.4			
58	19.45	[M-H]-	893.2313	603.1524 (51), 577.1377 (43), 451.1055 (88), 433.0938 (52), 425.0893 (46), 407.0784 (62), 315.0874 (56), 289.0724 (100)	-1.6	19.0	$C_{47}H_{42}O_{18}$	(epi)C-(epi)C-ethyl-(epi)C	3
59	19.57	[M-H]-	605.1675	453.1206 (9), 315.0882 (100), 289.0722 (94), 245.0829 (9), 203.0713 (10)	-1.8	13.6	$C_{32}H_{30}O_{12}$	(epi)C-ethyl-(epi)C	3
60	21.48	[M-H]-	389.1245	227.0712 (100), 185.0631 (6)	-0.7	19.0	$C_{20}H_{22}O_8$	piceid isomer	2
61	21.80	[M-H]-	893.2314	741.1884 (10), 603.1509 (50), 577.1350 (35), 451.1031 (71), 433.0934 (26), 425.0883 (34), 407.0779 (42), 315.0870 (84), 289.0710 (100)	-1.8	28.8	$C_{47}H_{42}O_{18}$	(epi)C-(epi)C-ethyl-(epi)C	3
62	22.14	[M-H]-	227.0705	227.0719 (100), 185.0612 (8), 143.0499 (2)	1.0	22.0	$C_{14}H_{12}O_3$	*trans*-resveratrol	1
63	23.42	[M-H]-	605.1657	453.1193 (8), 315.0872 (94), 289.0715 (100), 245.0819 (9), 203.0707 (10), 151.0385 (4)	1.2	9.9	$C_{32}H_{30}O_{12}$	(epi)C-ethyl-(epi)C	3
64	24.75	[M-H]-	207.0665	207.0662 (100), 179.0349 (11), 161.0238 (11), 135.0435 (10)	-1.2	1.3	$C_{11}H_{12}O_4$	ethyl caffeate / isomer	3
65	24.83	[M-H]-	227.0710	227.0708 (100), 185.0605 (24), 143.0495 (7)	1.8	9.6	$C_{14}H_{12}O_3$	resveratrol isomer	2
66	27.26	[M-H]-	191.0717	no fragmentation	-1.7	2.1	$C_{11}H_{11}O_3$	ethyl coumarate / isomer	4

Hex – hexose; HexA – hexuronic acid; (epi)C – (epi)catechin; (epi)GC – (epi)galocatechin. * – the level of confidence for the identification of identified compounds was evaluated according to Schymanski et al., 2014.

Table S4. Tentative identification of phenolics and other aromatic compounds in the 'Regent' wines.

No.	t_R	Ion type	*m/z*	Major fragment ions	Error [ppm]	mσ	Formula	Tentative identification	Identification level*
1	2.12	$[M-H]^-$	169.0140	169.0139 (100), 125.0243 (69)	1.2	3.3	$C_7H_6O_5$	gallic acid	1
2	3.41	$[M+H]^+$	166.0861	166.0859 (60), 149.0594 (4), 131.0494 (4), 120.0802 (100)	1.1	11.5	$C_9H_{11}NO_2$	phenylalanine / isomer	2
3	4.61	$[M-H]^-$	206.0450	206.0558 (98), 162.0556 (16), 146.0229 (64), 144.0437 (100), 134.0594 (42)	4.2	15.5	$C_{10}H_9NO_4$	unidentified aromatic acid	4
4	5.19	$[M-H]^-$	311.0407	311.0396 (10), 179.0339 (35), 149.0075 (100), 135.0436 (20)	0.4	29.7	$C_{13}H_{12}O_9$	caftaric acid isomer	2
5	5.24	$[M-H]^-$	457.0983	457.0980 (35), 325.0923 (93), 163.0385 (100), 119.0488 (49)	1.0	11.7	$C_{19}H_{21}O_{13}$	coutaric acid-Hex / isomer	3
6	5.36	$[M-H]^-$	593.1305	weak signal, no fragmentation	-0.8	20.9	$C_{30}H_{26}O_{13}$	(epiGC)-(epi)C	3
7	5.40	$[M-H]^-$	382.0993	382.0996 (100), 266.0879 (9), 250.0584 (7), 206.0675 (30), 162.0778 (6), 134.0450 (27)	-0.4	8.0	$C_{14}H_{17}N_5O_8$	succinyladenosine / isomer	2
8	5.50	$[M-H]^-$	315.1091	315.1092 (100), 153.0549 (42), 123.0455 (29)	-1.7	3.6	$C_{14}H_{20}O_8$	hydroxytyrosol hexoside / isomer	3
9	5.71	$[M-H]^-$	311.0405	179.0353 (100), 149.0084 (19), 135.0441 (46)	1.1	1.7	$C_{13}H_{12}O_9$	*trans*-caftaric acid	1
10	5.93	$[M-H]^-$	183.0290	183.0291 (100), 168.0055 (68), 124.0157 (47)	4.9	9.4	$C_8H_8O_5$	methylgallate / isomer	3
11	5.98	$[M-H]^-$	181.0498	181.0498 (100), 163.0388 (27), 135.0430 (22), 119.0487 (10)	4.6	2.3	$C_9H_{10}O_4$	hydroxyphenyllactic acid / isomer	2
12	6.11	$[M-H]^-$	616.1091	484.1040 (14), 466.0933 (16), 440.1140 (19), 343.0131 (2), 272.0891 (100), 254.0785 (11), 211.0072 (25), 192.9967 (12), 167.0173 (24), 149.0083 (13)	-0.1	12.0	$C_{23}H_{27}N_3O_{15}S$	caftaric acid-glutathione	2
		$[M+H]^+$	618.1240	618.1235 (100), 543.0919 (17), 489.0808 (11), 393.0753 (6), 386.0543 (12), 322.0384 (9), 264.0325 (25), 211.0713 (9), 195.0111 (10), 145.0606 (23)	-0.6	14.3			
13	6.31	$[M+H]^+$	205.0971	205.0970 (18), 188.0706 (100), 170.0603 (2), 159.0913 (4), 146.0599 (20), 132.0810 (2)	0.2	6.0	$C_{11}H_{12}N_2O_2$	tryptophan / isomer	2
14	6.35	$[M-H]^-$	517.1190	517.11951 (12), 355.0663 (100), 193.1034 (39)	1.8	5.5	$C_{21}H_{26}O_{15}$	unidentified dihexoside ($C_9H_6O_6$-Hex-Hex)	4
15	7.88	$[M-H]^-$	295.0462	295.0458 (7), 163.0398 (100), 149.0082 (73), 119.0496 (54)	-1.0	7.7	$C_{13}H_{12}O_8$	coutaric acid 1	3
16	8.10	$[M-H]^-$	295.0465	295.0468 (16), 163.0400 (100), 119.0502 (34)	-1.8	2.5	$C_{13}H_{12}O_8$	coutaric acid 2	3
17	8.20	$[M-H]^-$	577.1361	451.1041 (22), 425.0885 (37), 407.0776 (71), 289.0724 (100), 125.0242 (10)	-1.6	17.2	$C_{30}H_{26}O_{12}$	(epi)C-(epi)C	3
18	8.35	$[M-H]^-$	305.0655	low intensity & no reliable fragmentation	3.9	44.0	$C_{15}H_{14}O_7$	(epi)gallocatechin	2

19	8.41	$[M-H]^-$	577.1366	451.1057 (21), 425.0891 (47), 407.0785 (84), 289.0728 (100), 255.0305 (17), 125.0248 (12)	-2.5	4.8	$C_{30}H_{26}O_{12}$	(epi)C-(epi)C	3
20	8.79	$[M-H]^-$	289.0721	289.0722 (100), 245.0823 (19)	-1.2	13.3	$C_{15}H_{14}O_6$	catechin	1
21	9.20	$[M-H]^-$	589.0856	589.0864 (66), 509.1297 (100), 371.0753 (7), 347.0764 (37), 329.0664 (16), 314.0430 (11), 299.0196 (13)	2.2	14.4	$C_{23}H_{26}O_{16}S$	malvidin-Hex-SO_3H / isomer	3
22	9.37	M^+	641.1710	641.1704 (25), 479.1183 (14), 317.0658 (100)	0.4	12.9	$C_{28}H_{33}O_{17}$	petunidin-Hex-Hex	3
23	9.44	$[M-H]^-$	179.0351	179.0355 (100), 135.0441 (91)	-0.6	2.0	$C_9H_8O_4$	*trans*-caffeic acid*	1
24	9.66	$[M-H]^-$	325.0929	no MS/MS	-0.1	21.6	$C_{15}H_{18}O_8$	coumaric acid-Hex	3
25	9.68	$[M-H]^-$	229.0981	229.0977 (100), 185.1077 (29), 168.0805 (3), 142.0642 (7), 116.0490 (9)	0.6	5.6	$C_{13}H_{14}N_2O_2$	tetrahydroharman-3-carboxylic acid / isomer	3
26	9.78	$[M-H]^-$	451.1245	451.1257 (8), 289.0724 (100), 245.0820 (21), 221.0816 (10)	0.1	34.2	$C_{21}H_{24}O_{11}$	epicatechin-Hex / isomer	3
27	9.83	$[M-H]^-$	589.0866	589.0864 (77), 509.1295 (100), 371.0784 (6), 347.0767 (36), 329.0655 (12), 314.0425 (12), 299.0205 (17), 165.0187 (17)	0.5	24.4	$C_{23}H_{26}O_{16}S$	malvidin-Hex-SO_3H / isomer	3
28	10.10	$[M-H]^-$	613.1195	569.1307 (6), 417.0828 (8), 407.0770 (100), 255.0294 (72)	0.6	10.2	$C_{29}H_{26}O_{15}$	unidentified hexoside	4
		$[M+H]^+$	615.1347	615.1336 (14), 453.0810 (100), 435.0708 (14), 301.0341 (85), 283.0234 (4)	-0.3	6.8			
29	10.25	$[M-H]^-$	325.0563	193.0512 (100), 149.0076 (2), 134.0356 (31)	0.6	6.0	$C_{14}H_{14}O_9$	fertaric acid	3
30	10.31	$[M-H]^-$	865.1998	713.1498 (25), 695.1448 (13), 577.1362 (32), 575.1194 (29), 543.0940 (19), 451.1047 (46), 425.0882 (57), 407.0776 (61), 287.0568 (100), 243.0298 (34), 125.0239 (28)	-1.4	7.6	$C_{45}H_{38}O_{18}$	(epi)C-(epi)C-(epi)C	3
31	10.52	$[M-H]^-$	577.1349	451.1037 (16), 425.0892 (26), 407.0784 (48), 289.0725 (100), 125.0245 (8)	0.4	27.7	$C_{30}H_{26}O_{12}$	(epi)C-(epi)C	3
32	10.97	M^+	625.1751	625.1748 (28), 463.1227 (19), 301.0702	2.0	12.7	$C_{28}H_{33}O_{16}$	peonidin-Hex-Hex	2
33	11.32	M^+	655.1866	655.1853 (25), 493.1332 (13), 331.0805 (100)	0.4	14.7	$C_{29}H_{35}O_{17}$	malvidin-Hex-Hex	2
34	11.42	$[M-H]^-$	577.1345	577.1357 (18), 451.1047 (21), 425.0881 (44), 407.0776 (83), 289.0717 (100), 125.0240 (10)	1.2	10.3	$C_{30}H_{26}O_{12}$	procyanidin B2	1
35	12.29	$[M+H]^+$	673.1962	511.1428 (9), 493.1324 (2), 349.0905 (100), 331.0804 (7), 223.0592 (26), 195.0280 (6), 181.0487 (48)	1.9	15.4	$C_{29}H_{36}O_{18}$	dihydrosyringetin-Hex-Hex / isomer	3
		$[M-H]^-$	671.182	509.1301 (100), 463.1251 (9), 371.0768 (7), 347.0772 (42), 329.0669 (33), 314.0428 (21), 299.0428 (21), 165.0184 (9)	1.3	7.5	$C_{29}H_{36}O_{18}$		
36	12.53	$[M-H]^-$	289.0717	289.0718 (100), 245.0815 (21), 221.0819 (7)	0.1	2.2	$C_{15}H_{14}O_6$	epicatechin	1
37	12.99	$[M-H]^-$	163.0396	163.0396 (53), 119.0495 (100)	2.8	7.6	$C_9H_8O_3$	*trans*-*p*-coumaric acid	1
38	13.02	$[M-2H]^{2-}$	576.1285	no fragmentation	0.9	79.8	$C_{60}H_{48}O_{24}$	(epi)C-(epi)C-(epi)C-(epi)C	4

39	13.37	[M-H]$^-$	197.0451	197.0451 (100), 169.0141 (14), 124.0160 (22)	1.3	5.4	$C_9H_{10}O_5$	ethyl gallate / isomer	3
40	13.37	[M+FA-H]$^-$	701.1935	493.1351 (47), 461.1096 (5), 331.0821 (100)	-0.1	2.6	$C_{29}H_{36}O_{17}$	dimethyltaxifolin-Hex-Hex / isomer	3
41	13.53	[M-H]$^-$	427.0345	427.0342 (82), 347.0770 (100), 345.0616 (23), 261.0757 (13), 165.0179 (10)	-1.1	12.3	$C_{17}H_{16}O_{11}S$	malvidin-SO_3H / isomer	3
42	13.69	[M-H]$^-$	366.1190	366.1192 (100), 204.0664 (9), 186.0559 (7), 158.0601 (2), 142.0650 (8)	1.2	4.2	$C_{17}H_{21}NO_8$	indolyllactic acid-Hex / isomer	3
43	13.80	[M-H]$^-$	465.1038	465.1043 (49), 339.0725 (7), 313.0926 (5), 303.0506 (6), 285.0405 (100), 151.0025 (15)	0.2	19.4	$C_{21}H_{22}O_{12}$	taxifolin-Hex / isomer	3
44	14.81	M$^+$	945.2630	793.2168 (13), 783.2112 (11), 631.1641 (21), 621.1587 (29), 603.1483, 495.1275 (32), 469.1118 (100), 451.1012 (21), 343.0804 (63), 331.0805 (27)	3.1	25.0	$C_{44}H_{49}O_{23}$	malvidin-(3-*O*-Hex)-(Epi)C-Hex [A type]	3
45	14.91	M$^+$	547.1065	547.1068 (15), 385.0545 (100)	3.1	14.9	$C_{25}H_{23}O_{14}$	vinylformic acid adduct of petunidin 3-*O*-Hex	3
46	15.43	[M+H]$^+$	206.0805	206.0806 (100), 188.0701 (51), 170.0595 (14), 160.0752 (31), 146.0596 (13), 130.0653 (12), 118.0646 (6)	3.5	6.6	$C_{11}H_{11}NO_3$	indolellactic acid	2
		[M-H]$^-$	204.0662	186.0558 (12), 158.0599 (19), 142.0659 (3), 128.0495 (5), 116.0499 (4)	2.3	10			
47	15.54	M$^+$	493.1340	493.1336 (22), 331.0809 (100)	0.2	12.6	$C_{23}H_{25}O_{12}$	malvidin -Hex	2
		[M-2H]$^-$	491.1187	491.1190 (13), 329.0660 (100), 313.0355 (72), 299.0199 (33)	1.6	12.0			
		[M+H_2O-2H]$^-$	509.1294	355.0674 (48), 347.0563 (84), 329.06273 (100), 314.0431 (25), 299.0198 (94), 217.0252 (37)	1.4	10.6			
48	15.86	M$^+$	697.1957	697.1961 (27), 535.1437 (15), 493.1331 (2), 331.0808 (100)	2.5	48.5	$C_{31}H_{37}O_{18}$	malvidin -Hex-AcA-Hex	3
49	16.15	[M-2H]$^-$	943.2494	781.1091 (100), 583.1257 (4), 493.1123 (3), 407.0772 (7)	2.1	10.7	$C_{44}H_{49}O_{23}$	malvidin-(3-O-Hex)-(Epi)C-Hex [A type]	3
50	17.29	[M-H]$^-$	389.1234	no fragmentation	2.0	8.1	$C_{20}H_{22}O_8$	piceid	1
51	17.60	M$^+$	561.1240	561.1237 (16), 399.0710 (100)	-0.2	20.9	$C_{26}H_{25}O_{14}$	vitisin A (pyranoanthocyanin) / isomer	3
		[M-2H]$^-$	559.1092	515.1200 (3), 353.0659 (100), 337.0348 (46), 323.0194 (80), 295.0239 (46)	0.2	18.3			
52	18.44	M$^+$	517.1335	517.1336 (22), 355.0811 (100)	0.7	14.9	$C_{25}H_{25}O_{12}$	vitisin B (pyranoanthocyanin) / isomer	3
53	18.83	[M+H]$^+$	479.0820	479.0814 (12), 303.0501 (100)	0.1	9.2	$C_{21}H_{18}O_{13}$	quercetin-HexA	3
		[M-H]$^-$	477.0675	477.0669 (17), 301.0349 (100), 255.0288 (10)	0.0	16.2			
54	18.93	M$^+$	971.2793	971.2795 (29), 809.2275 (6), 681.2275 (7), 647.1753 (6),	2.3	28.8	$C_{46}H_{51}O_{23}$	malvidin-3-O-Hex-8-ethyl-	3

				519.1492 (55), 495.1280 (2), 357.0966 (100)				(epi)cat-Hex	
55	18.99	[M+H]+	451.123	451.1236 (7), 305.0659 (100), 287.0554 (14), 259.0602 (18)	1.1	9.4	$C_{21}H_{22}O_{11}$	taxifolin-dHex / isomer	3
		[M-H]-	449.1079	449.1087 (89), 303.0510 (56), 287.1489 (58), 285.0403 (100), 269.0442 (36)	2.3	23.4			
56	18.99	[M+H]+	819.2327	801.2227 (6), 657.1812 (10), 639.1701 (13), 511.1441 (7), 493.1339 (3), 349.0918 (100), 331.0812 (32), 309.0972 (21), 223.0599 (45), 181.0494 (30), 147.0437 (31)	1.8	14.9	$C_{38}H_{42}O_{20}$	dihydrosyringetin-Hex-CouA-Hex / isomer	3
57	19.56	M+	971.2810	971.2800 (28), 809.2281 (4), 681.2028 (8), 647.1754 (3), 519.1500 (55), 495.1291 (2), 357.0972 (100)	0.6	25.9	$C_{46}H_{51}O_{23}$	malvidin-3-O-Hex-8-ethyl-(epi)cat-Hex	3
58	19.72	M+	817.2182	817.2180 (19), 655.1657 (8), 493.1341 (4), 331.0816 (100)	0.5	39.5	$C_{38}H_{41}O_{20}$	malvidin -Hex-CafA-Hex	3
59	19.94	M+	787.2080	787.2073 (23), 625.1539 (8), 479.1193 (4), 317.0660 (100)	1.1	9.1	$C_{37}H_{39}O_{19}$	petunidin-Hex-Hex-CouA	3
60	20.36	[M-2H]-	807.2141	645.1605 (100), 517.1349 (50), 491.1215 (45), 355.0823 (83), 329.0664 (99), 315.0880 (87)	0.1	38.6	$C_{40}H_{41}O_{18}$	malvidin-3-*O*-Hex-8-ethyl-(epi)cat	3
		M+	809.2289	647.1753 (2), 519.1492 (7), 495.1286 (2), 357.0969 (100)	0.5	11.6			
61	20.73	[M-2H]-	807.2125	645.1598 (93), 517.1351 (31), 491.1194 (27), 355.0812 (65), 329.0661 (100), 315.0868 (74)	2.0	25.0	$C_{40}H_{40}O_{18}$	malvidin-3-*O*-Hex-8-ethyl-(epi)cat	3
		M+	809.2291	809.2288 (26), 647.1758 (4), 519.1503 (7), 495.1287 (3), 357.0973 (100)	-0.4	15.0			
62	21.14	M+	801.2225	801.2226 (19), 639.1708 (8), 439.1342 (3), 331.0815 (100)	1.4	8.6	$C_{38}H_{41}O_{19}$	malvidin-Hex-Hex-CouA	3
63	21.48	[M-H]-	389.1245	227.0712 (100), 185.0631 (6)	-0.7	19	$C_{20}H_{22}O_{8}$	piceid isomer	2
64	22.01	[M-H]-	507.1139	507.1143 (100), 344.0534 (24), 329.0297 (20), 315.0143 (9), 301.0352 (37)	1.0	4.0	$C_{23}H_{23}O_{13}$	syringetin-Hex	3
		[M+H]+	509.1292	347.0766 (100)	-0.4	15.2			
65	22.05	M+	707.1607	707.1602 (23), 399.0715 (100)	-0.1	21.8	$C_{35}H_{31}O_{16}$	vitisin A-CouA / isomer	3
66	22.14	[M-H]-	227.0705	227.0719 (100), 185.0612 (8), 143.0499 (2)	1.0	22.0	$C_{14}H_{12}O_{3}$	*trans*-resveratrol	1
67	22.51	[M-H]-	1089.2851	927.2352 (100), 801.2040 (6), 781.2000 (6), 601.1346 (3), 583.1241 (12), 493.1134 (27), 455.0552 (7), 403.0456 (40), 335.0558 (16)				unidentified	5
68	23.30	M+	805.1964	805.1973 (35), 643.1444 (100), 491.0976 (31)	1.3	38.4	$C_{40}H_{37}O_{18}$	malvidin-3-*O*-Hex-4-vinyl(epi)cat	3
69	23.67	M+	595.1442	595.1441 (34), 433.0912 (100)	0.6	15.5	$C_{30}H_{27}O_{13}$	petunidin 3-*O*-Hex-4-vinylphenol / isomer	3
		[M-2H]-	593.1305	430.0699 (100), 415.0458 (45), 387.0505 (76), 359.0560 (61)	-0.7	18.1			
70	23.71	M+	639.1708	639.1703 (23), 331.0811 (100)	0.9	7.5	$C_{32}H_{31}O_{14}$	malvidin-Hex-CouA	3

71	23.73	M^{+}	955.2642	955.2643 (21), 665.1862 (8), 647.1754 (2), 357.0968 (100), 331.0818 (2)	1.3	16.9	$C_{49}H_{47}O_{20}$	malvidin-3-*O*-Hex-CouA-8-ethyl-(epi)cat	3
		[M-2H]$^{-}$	953.2503	663.1708 (49), 645.1615 (100), 637.1564 (47), 355.0821 (71), 339.0501 (57), 329.662 (75), 315.0869 (52),289.0711 (35)	0.7	31.7			
72	24.22	M^{+}	805.1958	805.1957 (45), 643.1437 (100), 491.0964 (26)	2.1	23.0	$C_{40}H_{37}O_{18}$	malvidin-3-*O*-Hex-4-vinyl(epi)cat	3
73	24.75	[M-H]$^{-}$	207.0665	207.0662 (100), 179.0349 (11), 161.0238 (11), 135.0435 (10)	-1.2	1.3	$C_{11}H_{12}O_{4}$	ethyl caffeate / isomer	3
74	24.78	M^{+}	609.1585	609.1587 (28), 447.1064 (100)	2.9	5.6	$C_{31}H_{29}O_{13}$	malvidin 3-*O*-Hex-4-vinylphenol / isomer	3
		[M-2H]$^{-}$	607.1454	607.1435 (10), 445.0917 (100), 429.0620 (20), 415.0455 (27), 386.0427 (57), 358.0477 (39)	0.5	48.9			
75	24.80	[M-H]$^{-}$	227.0709	227.0709 (100), 185.0605 (20), 143.0486 (4)	2.1	12.9	$C_{14}H_{12}O_{3}$	resveratrol isomer	2
76	25.10	M^{+}	639.1692	639.1693 (29), 477.1165 (100)	2.6	22.5	$C_{32}H_{31}O_{14}$	malvidin-3-*O*-Hex-4-vinylguaiacol	3
77	25.20	M^{+}	955.2633	955.2627 (17), 665.1852 (8), 647.1742 (2), 495.1284 (2), 357.0960 (100), 331.0802 (2)	2.4	47.0	$C_{49}H_{47}O_{20}$	malvidin-3-*O*-Hex-CouA-8-ethyl-(epi)cat	3
78	25.20	[M+H]$^{+}$	197.0803	197.0800 (100), 169.0488 (78), 151.0385 (18), 125.0593 (35)	2.7	15.9	$C_{10}H_{12}O_{4}$	ethyl vanillate / isomer	3
79	25.41	[M+H]$^{+}$	227.0907	227.0904 (100), 199.0594 (27), 181.0486 (30), 155.0695 (75), 140.0458 (55), 123.0438 (12)	3.1	2.6	$C_{11}H_{14}O_{5}$	ethyl syringate / isomer	3
80	26.15	M^{+}	755.1955	755.1943 (22), 447.1059 (100)	2.1	10.4	$C_{40}H_{35}O_{15}$	malvidin 3-*O*-Hex-CouA-4-vinylphenol / isomer	3
81	27.27	[M-H]$^{-}$	191.0712	no fragmentation	0.6	3.7	$C_{11}H_{11}O_{3}$	ethyl coumarate / isomer#	4

Hex – hexose; HexA – hexuronic acid; dHex – deoxyhexose; AcA – acetic acid; CafA – caffeic acid; CouA – coumaric acid; (epi)C – (epi)catechin; (epi)GC – (epi)gallocatechin; * – the level of confidence for the identification of compounds was evaluated according to Schymanski et al., 2014; # – a clear UV spectrum available to confirm identification;

Reference:

Schymanski, E.L., Jeon, J.; Gulde, R.; Fenner, K., Ruff, M.; Singer, H.P.; Hollender, J. Identifying small molecules via high resolution mass spectrometry: communicating confidence. *Environ. Sci. Technol.* **2014**, *48*, 2097–2098. https://doi.org/10.1021/es5002105

Table S5. Antioxidant capacities of the 'Johanniter' and 'Regent' wines determined by the FRAP assay.

**Maceration time** (days)	**Antioxidant capacity (FRAP)** (TE; mmol/100 mL)	
	**'Johanniter'**	**'Regent'**
**0**	**3.97 ± 0.23 a**	**4.77 ± 0.36 b**
4	13.50 ± 0.04 d	10.39 ± 0.01 c
8	14.49 ± 0.23 e	13.52 ± 0.06 d
12	14.82 ± 0.26 e	13.87 ± 0.14 d
16	16.27 ± 0.08 g	13.86 ± 0.31 d
20	16.58 ± 0.01 g	15.56 ± 0.08 f
Average	13.27 ± 4.42 B	12.00 ± 3.69 A

Values marked with different letters differ significantly in a Tukey's post hoc test, assuming a significance threshold $p < 0.01$ to indicate highly significant differences between sample parameters. The lowercase letters (a–g) indicate differences across all data; capital letters (A, B) indicate significance of differences for average values. Data are presented as mean values ± SD; n = 3.

Table S6. Antiradical capacities of the 'Johanniter' and 'Regent' wines determined by the DPPH colorimetric assay.

**Maceration time** (days)	**Antiradical capacity (DPPH)** (TE; mmol/100 mL)	
	**'Johanniter'**	**'Regent'**
**0**	**0.08 ± 0.01 a**	**0.20 ± 0.01 b**
4	0.52 ± 0.04 d	0.33 ± 0.01 c
8	0.54 ± 0.03 d	0.33 ± 0.02 c
12	0.56 ± 0.03 d	0.33 ± 0.01 c
16	0.54 ± 0.02 d	0.34 ± 0.01 c
20	0.54 ± 0.03 d	0.34 ± 0.01 c
Average	0.46 ± 0.18 B	0.31 ± 0.05 A

Values marked with different letters differ significantly in a Tukey's post hoc test, assuming a significance threshold at $p < 0.05$. The lowercase letters (a–d) indicate differences across all data; capital letters (A, B) indicate significance of differences for average values. Data are presented as mean values ± SD; n = 3.

Table S7. Antiradical capacities of the 'Johanniter' and 'Regent' wines determined by the ORAC-fl assay.

**Maceration time** (days)	**Antiradical capacity (ORAC-fl)** (TE; mmol/100 mL)	
	**'Johanniter'**	**'Regent'**
**0**	**1.79 ± 0.33 a**	**1.70 ± 0.95 a**
4	4.76 ± 1.03 b	7.46 ± 0.53 bcde
8	5.68 ± 0.96 bc	8.25 ± 1.08 cde
12	5.95 ± 1.10 bc	9.01 ± 1.13 de
16	6.47 ± 0.77 bcd	8.26 ± 1.09 cde
20	6.66 ± 0.84 bcde	9.42 ± 1.20 e
Average	5.22 ± 1.85 A	7.35 ± 2.82 B

Values marked with different letters differ significantly in a Tukey's post hoc test, assuming a significance threshold $p < 0.01$ to indicate highly significant differences between sample parameters. The lowercase letters (a–e) indicate differences across all data; capital letters (A, B) indicate significance of differences for average values. Data are presented as mean values ± SD; n = 3.
